# Novel 4-Azapregnene Derivatives as Potential Anticancer Agents: Synthesis, Antiproliferative Activity and Molecular Docking Studies

**DOI:** 10.3390/molecules27186126

**Published:** 2022-09-19

**Authors:** Vanessa Brito, Adriana Oliveira Santos, Gilberto Alves, Paulo Almeida, Samuel Silvestre

**Affiliations:** 1 Health Sciences Research Centre (CICS-UBI), Universidade da Beira Interior, Av. Infante D. Henrique, 6200-506 Covilhã, Portugal; 2 Center for Neuroscience and Cell Biology (CNC), University of Coimbra, 3004-517 Coimbra, Portugal

**Keywords:** 4-azasteroids, 4-azapregnenes, aldol condensation, molecular docking, antiproliferative activity

## Abstract

A series of novel 21*E*-arylidene-4-azapregn-5-ene steroids has been successfully designed, synthesized and structurally characterized, and their antiproliferative activity was evaluated in four different cell lines. Within this group, the 21*E*-(pyridin-3-yl)methylidene derivative exhibited significant cytotoxic activity in hormone-dependent cells LNCaP (IC_50_ = 10.20 µM) and T47-D cells (IC_50_ = 1.33 µM). In PC-3 androgen-independent cells, the steroid 21*E*-*p*-nitrophenylidene-4-azapregn-5-ene was the most potent of this series (IC_50_ = 3.29 µM). Considering these results, the 21*E*-(pyridin-3-yl)methylidene derivative was chosen for further biological studies on T47-D and LNCaP cells, and it was shown that this azasteroid seems to lead T47-D cells to apoptotic death. Finally, molecular docking studies were performed to explore the affinity of these 4-azapregnene derivatives to several steroid targets, namely 5α-reductase type 2, estrogen receptor α, androgen receptor and CYP17A1. In general, compounds presented higher affinity to 5α-reductase type 2 and estrogen receptor α.

## 1. Introduction

Natural, semi-synthetic and synthetic steroidal derivatives are a rich source of potential drug candidates, being a current focus of investigation [1,2,3]. In addition, steroids exhibit several biological advantages, such as a low toxicity, less vulnerability to multi-drug resistance and high bioavailability [4,5,6]. Therefore, a large number of modified steroids have been described, principally, as potential antitumor agents [7,8]. For example, some 17(*E*)-picolinylidene androstane derivatives were reported as potential inhibitors of prostate and breast cancer cell growth. Interesting antiproliferative activity against prostate PC-3 cells was observed and this effect is correlated with the cytotoxic effect observed for abiraterone, a drug with clinical use in prostate cancer (PCa) treatment [9].

Among other explored chemical modifications, the insertion of a 16-arylidene group into the steroid structure has also been associated with significant cytotoxic effects in several cell lines, being considered a relevant pharmacophore for anticancer activity [10,11,12,13]. Moreover, several pregnane derivatives have emerged as potential antitumor agents, showing relevant results in this context [5,6,14,15,16,17,18]. Therefore, considering this, Banday et al. reported the synthesis of novel benzylidene pregnenolone derivatives and their antiproliferative effects against a panel of human cancer cell lines. This research showed interesting results, with some of these steroids presenting very potent effects especially against colon (HCT-15) and breast (MCF-7) cancer cells [16].

Other semi-synthetic steroidal derivatives widely explored over the years are 4-azasteroids, such as finasteride and dutasteride, which are clinically used to treat benign prostatic hyperplasia (BPH), inhibiting the conversion of testosterone to 5α-dihydrotestosterone (DHT) by 5α-reductase (5AR) enzymes [7]. In addition, 5AR inhibitors have been evaluated for their potential use as chemopreventive agents against PCa, since all isoforms of this enzyme are present in PCa at increased levels. Moreover, different 4-azasteroids are also being explored as anticancer agents. For example, a research study reported the synthesis of 4-azasteroidal purine nucleoside analogs and their evaluation as antitumor agents in MCF-7 and PC-3 cell lines, and some of these derivatives revealed potent antiproliferative activity against PCa cells [19]. Recently, we also described the synthesis, biological evaluation and in silico studies of novel 16-arylidene-4-azaandrostenes with antiproliferative effects, and interesting results were observed in prostatic cancer cell lines. In addition, a significant selectivity towards cancer cell lines was found for all azaandrostenes, presenting low cytotoxicity in non-cancerous human fibroblasts. Moreover, the molecular docking studies showed that these 4-azaandrostene derivatives can interact with 5AR type 2, as well as with other common targets of steroidal drugs [20].

Based on the aforementioned studies and intending to develop compounds with higher potency and selectivity, the present work foucuses on the preparation of new 21-arylideneazapregnene derivatives and the biological evaluation of their effects in several cell lines, such as prostatic (LNCaP, PC-3) and breast (T47-D) cancer cells, and on non-cancerous dermal fibroblasts. The structure of the synthesized steroids was validated through adequate structural characterization techniques. The cytotoxic effects evaluation of the novel arylidenes was performed by the 3-(4,5-dimethylthiazol-2-yl)-2,5-diphenyltetrazolium bromide (MTT) assay. Moreover, the antiproliferative effects in T47-D and LNCaP cells for the most relevant steroids were further explored by different cellular and molecular biology techniques. Furthermore, in silico molecular docking simulations against important targets of steroidal molecules were performed [8,9,21,22,23]. 

## 2. Results and Discussion

### 2.1. Chemistry

**Scheme 1:**
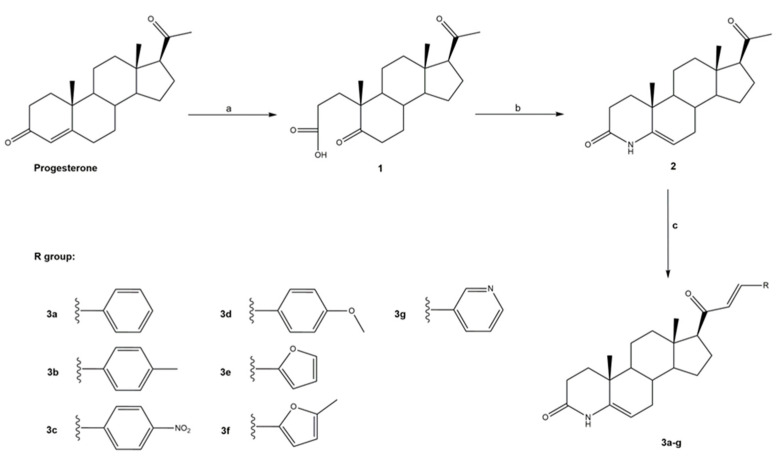
Synthesis of the steroids **3a**–**g** from progesterone. Reagents, reaction conditions and yields: (a) NaIO_4_, KMnO_4_, Na_2_CO_3_, *i*-PrOH, reflux 3 h, 89%; (b) CH_3_COONH_4_, CH_3_COOH, reflux 4 h, 98%; (c) KOH, aldehyde, EtOH, r.t., 12–24 h, 46–84%.

The synthesis of 4-azapregn-5-ene-3,20-dione derivatives has been carried out as depicted in Scheme 1. Progesterone was treated with sodium periodate and potassium permanganate to form **1**, by an oxidative cleavage reaction, with an excellent yield of 89%. Then, 4-azapregn-4-ene-3,20-dione (**2**) was obtained from an azacyclization reaction of **1**. Distinct procedures for this reaction with different catalysts or reagents and energy sources are described [24,25]. Within these methods, the use of acetic acid and ammonium acetate proved to be a practical approach and allowed for the preparation of the desired product with a high yield, but the removal of acetic acid revealed to be a difficult task. To overcome this issue, extraction with a larger portion of dichloromethane (DCM), followed by washing the organic layer with brine three times, to ensure the total removal of the acetic acid present in the reactional mixture, was required. Infrared (IR) and nuclear magnetic resonance (NMR) spectroscopy data of **1** and **2** were similar to the described in the literature [25].

The changes on the side chain of steroids are relevant since these modifications frequently lead to more effective receptor binding or increased bioavailability [15]. Taking into account the cytotoxicity against tumor cells associated with structural analogs, such as 16*E*-arylidene-4-azaandrostene derivatives, it was decided to synthesize novel 4-azapregnene derivatives with modifications on the side chain by introducing an arylidene group [17,20,26,27,28]. Particularly, the 4-azaandrostene derivatives reported by us presented some interesting cytotoxicity results, including a relevant selectivity toward cancer cell lines since, generally, low cytotoxicity was detected in non-cancerous human fibroblasts. Furthermore, these steroids induced a reduction of viability in LNCaP cells comparable to the observed with finasteride [20]. Similarly, in the present work, an aldol condensation of 4-azapregn-4-ene-3,20-dione with several aldehydes at room temperature in alkaline medium afforded the corresponding arylidene derivatives **3a**–**g** in very acceptable yields, as shown in Table 1 [11,13]. The ^1^H NMR spectra revealed, typically, the signals of the vinylic protons, being 21-H and 21a-H at ≈7.47 and ≈6.70 ppm, respectively. An observed coupling constant of 16.0 Hz unequivocally indicated the *E* geometry for the double bond, which is in agreement with the literature [17,18,29].

### 2.2. Biology

#### 2.2.1. Screening of Cell Proliferation Effects 

The effects of steroids **3a**–**g** and synthetic precursors (progesterone, **1** and **2**) on the proliferation of LNCaP, PC-3, T47-D and normal human dermal fibroblasts (NHDF) cells were examined by the MTT proliferation assay. These cell lines were used as models of androgen-dependent and androgen-independent prostate cancer, hormone-responsive breast cancer and non-cancerous cells, respectively, to evaluate the selective cytotoxicity of these compounds. Due to the structural similarities between the prepared azasteroidal derivatives and finasteride, this drug was also included in the study, as well as testosterone and DHT, allowing to compare the effect on cell proliferation in relation to the novel arylidenes herein described. Additionally, 5-fluorouracil (5-FU), a clinically used antitumor agent, was also included in the assay as a positive control. Firstly, cells were exposed to all compounds at 30 μM, during 72 h, in a screening assay, similarly to which was performed by other authors [30,31,32]. Following this preliminary evaluation, concentration–response studies were performed for the most active derivatives and 5-FU in all cell lines, and the half-maximal inhibitory concentration (IC_50_) of the tested compounds was determined. Screening and IC_50_ results are shown in Table 2. The IC_50_ determination was performed for arylidenes **3a**, **3c** and **3g** in all cell lines, and **3b** in T47-D and PC-3 cells. Interestingly, the steroid **3g** was the most potent in two of the three tumor cell lines, with an IC_50_ of 10.20 μM in LNCaP cells and 1.33 μM in T47-D cells. In PC-3 cell line, derivatives **3c** and **3g** presented very close IC_50_ values, 3.29 μM and 3.64 μM, respectively. Considering these results, azasteroids **3c** and **3g** seemed promising candidates for more advanced studies, despite the relevant cytotoxicity of **3g** against the non-cancerous cells.

#### 2.2.2. Characterization of the Cytotoxic Effect of **3g** on T47-D Cells

**Figure 1:**
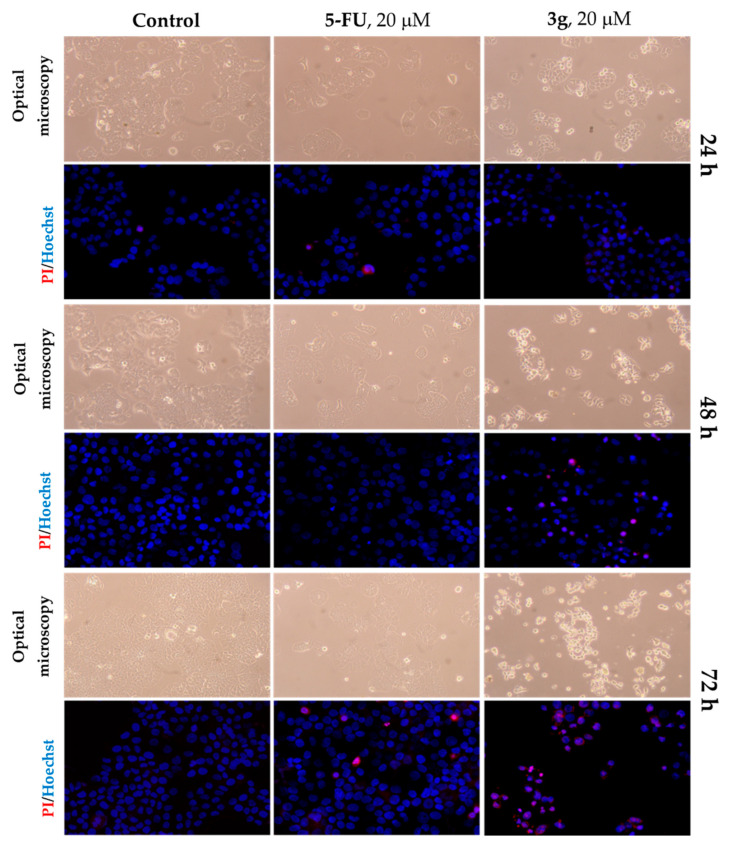
PI/Hoechst 3342 staining results. T47-D cells were incubated for 24, 48 and 72 h with 5-fluorouracil (5-FU) and **3g** at 20 µM. Untreated cells were considered the control of the experiment. Cells were visualized in an Optical microscope Olympus CKX41 coupled to a digital camera (Olympus SP-500UZ) and in an Axio Imager A1 microscope (fluorescence). The resulting images were treated in ImageJ software.

**Figure 2:**
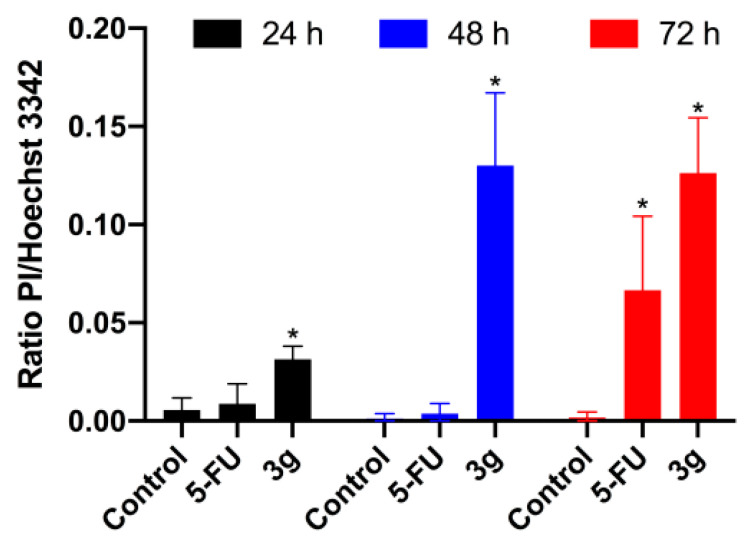
Ratio of PI/Hoechst 3342 staining results. T47-D cells were incubated during 24, 48 and 72 h with 5-fluorouracil (5-FU) and **3g** at 20 µM. Untreated cells were considered the control of the experiment. Cells were visualized in an Axio Imager A1 microscope, and the resulting images were treated in ImageJ. Data are expressed as mean ± SD and represent at least two experiments. * *p* < 0.05 compared to control.

The 21*E*-(pyridin-3-yl)methylidene derivative **3g**, was the most potent of this series of steroids on T47-D cells, with a IC_50_ of 1.33 μM. In order to better evaluate the cytotoxic effect of this compound, a fluorescence microscopy study by staining cells with Hoechst 3342—a nucleus marker—and PI—a cell death marker—was performed. For this, after 24, 48 and 72 h of treatment with **3g** at 20 μM, cells were observed through optical microscopy and then stained for fluorescence microscopy observation. Cells exposed to **3g** had a more frequent PI staining than positive and negative controls (Figure 1), as evidenced by the quantification of a higher ratio of PI/Hoechst 3342 staining (Figure 2). These results indicate that cells incubated with **3g** are dying with increased frequency from an early time point after treatment when compared to negative and positive controls.

**Figure 3:**
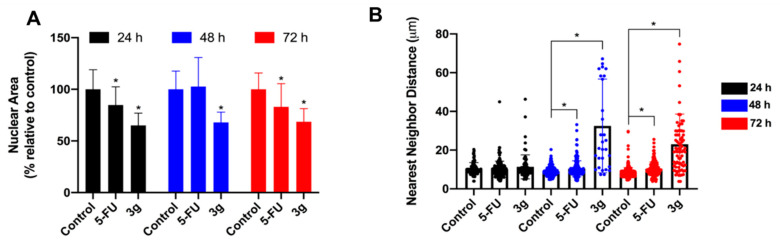
(**A**) T47D nuclear area of untreated cells (control) and cells treated with 5-fluorouracil (5-FU) and **3g** at 20 µM, incubated 24, 48 and 72 h; (**B**) nearest neighbor analysis of T47-D cells with the same conditions referred previously. Data are mean ± SD (**A**) or individual points ± SD (**B**) and represent at least two experiments. * *p* < 0.05 compared to control.

Several morphological modifications occurred over time when cells were exposed to **3g** and, also, to 5-FU, being more evident when cells were treated with the azasteroid (Figure 1). These were mainly cell rounding and the decrease of the cells’ area and volume, which were quantified with ImageJ software—Figure 3A. In this context, the **3g**-incubated cultures showed a significant decrease in the nuclear area after all incubation times. The decrease in cell nuclei area due to DNA condensation/loss is an indicator of apoptosis, and this correlation was validated by Eidet et al. (2014) [33]. The nearest neighbor analysis (NND) of T47-D cells when treated with the testing compound is shown in Figure 3B. The NND is measured to assess the cell nuclei distribution, and is defined as the distance between the centroid of each nucleus and its closest one. This analysis was employed to evaluate if the treatment (5-FU and **3g**) causes changes in cell distribution. Moreover, cells treated for 48 and 72 h with 5-FU and **3g** displayed different distances, which means a more aleatory placement of the cells. The literature points out that these results are in agreement with the occurrence of apoptosis, which seems to cause a more unequal cell spacing [33]. 

**Figure 4:**
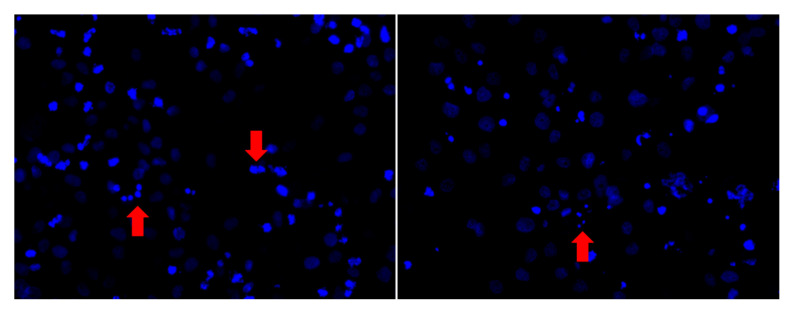
T47-D cells stained with Hoechst 3342, after treatment with **3g** during 72 h. Pycnotic cells with circular morphology are indicative of apoptosis.

Lastly, nuclei with a morphology coincidental with apoptotic cells are indicated with red arrows, and they are presenting a circular and pycnotic form (Figure 4).

**Figure 5:**
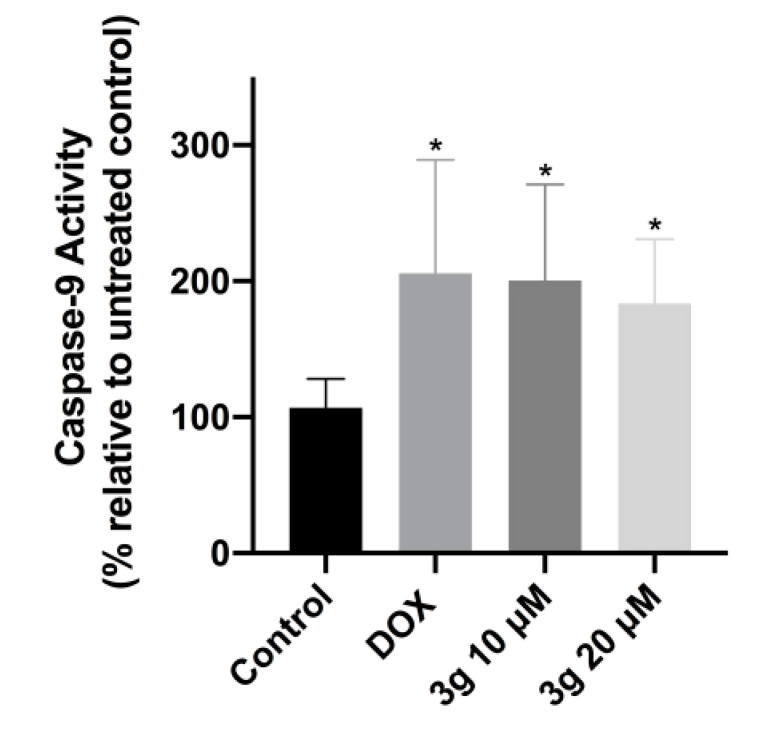
T47D caspase-9 activity results. Cells were treated for 48 h with doxorubicin (DOX) at 10 µM, as the positive control, and **3g** at 10 and 20 µM, and the results are relative to untreated cells (control). The activity of caspase-9 was evaluated using a Caspase-Glo kit from Promega, as described in Material and Methods. Data are expressed as mean ± SD (2 independent assays, n = 4 in each). * *p* < 0.05 compared to control.

To explore the possible mechanism of apoptotic death in T47-D cells, the caspase-9 activity was measured using a Caspase-Glo kit from Promega looking to consolidate caspase-dependent apoptosis. It is known that caspase-9 has an important role in the intrinsic or mitochondrial pathway of the apoptosis death mechanism [34]. In this experiment, cells were exposed for 48 h to doxorubicin (DOX) at 10 µM, as the positive control, and to **3g** at 10 and 20 µM, and the results obtained are shown in Figure 5. After 48 h of treatment, significant elevation in caspase-9 activity was observed in cells treated with DOX and with both concentrations of **3g**, which is a relevant indication that this steroid triggers the apoptotic mechanism of cell death.

#### 2.2.3. Characterization of the Cytotoxic Effect of **3g** on LNCaP Cells

**Figure 6:**
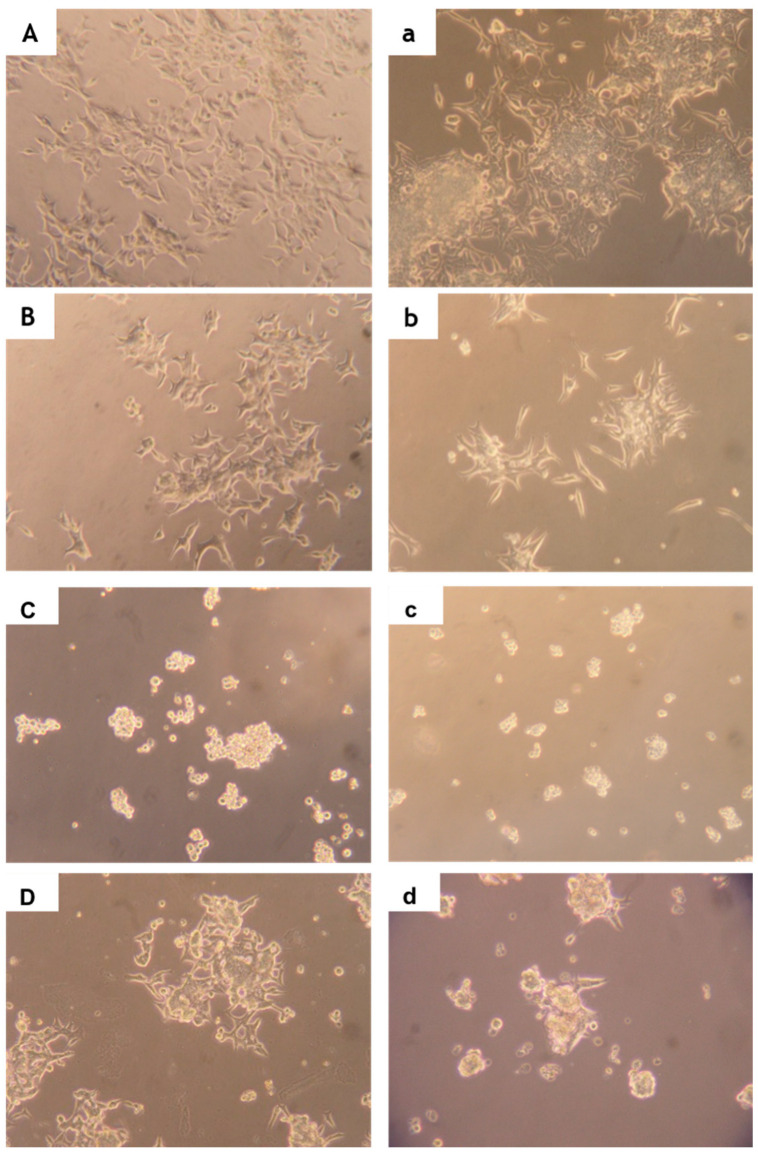
Photograph shots after incubation of LNCaP cells with the compounds (24 and 72 h, zoom: 100×). (**A**) Control after 24 h, (**a**) control after 72 h; (**B**) cells after 24 h of incubation with finasteride, (**b**) cells after 72 h of incubation with finasteride; (**C**) cells after 24 h of incubation with **3g**, (**c**) cells after 72 h of incubation with **3g**; and (**D**) cells after 24 h of incubation with 5-fluorouracil (5-FU), (**d**) cells after 72 h of incubation with 5-FU.

In our previous work, 16*E*-arylidene-4-azaandrost-5-ene derivatives were synthesized, their antiproliferative effects were evaluated and further studies were performed with the most promising steroid in LNCaP cells [20]. Due to the similarity of 16*E*-arylidene-4-azaandrostenes and these novel 21*E*-arylidene-4-azapregnene derivatives, identical studies in this cell line were performed, namely a flow cytometry assay and microscopic cell morphology observation. To assess the viability of LNCaP cells when exposed to steroid **3g**, a flow cytometry assay after PI staining was performed. Cells were treated with **3g** and 5-FU during 24 and 72 h, and then a microscopic cell observation was performed to assess the possible morphological alterations and cell density changes triggered by incubation with **3g** and compare with controls (Figure 6). A cell density reduction and the preservation of healthy cells morphology, when treated with finasteride, was observed. In contrast, cells treated with **3g**, after 24 and 72 h of treatment, appeared as detached, forming clusters and presenting a round form. 

**Figure 7:**
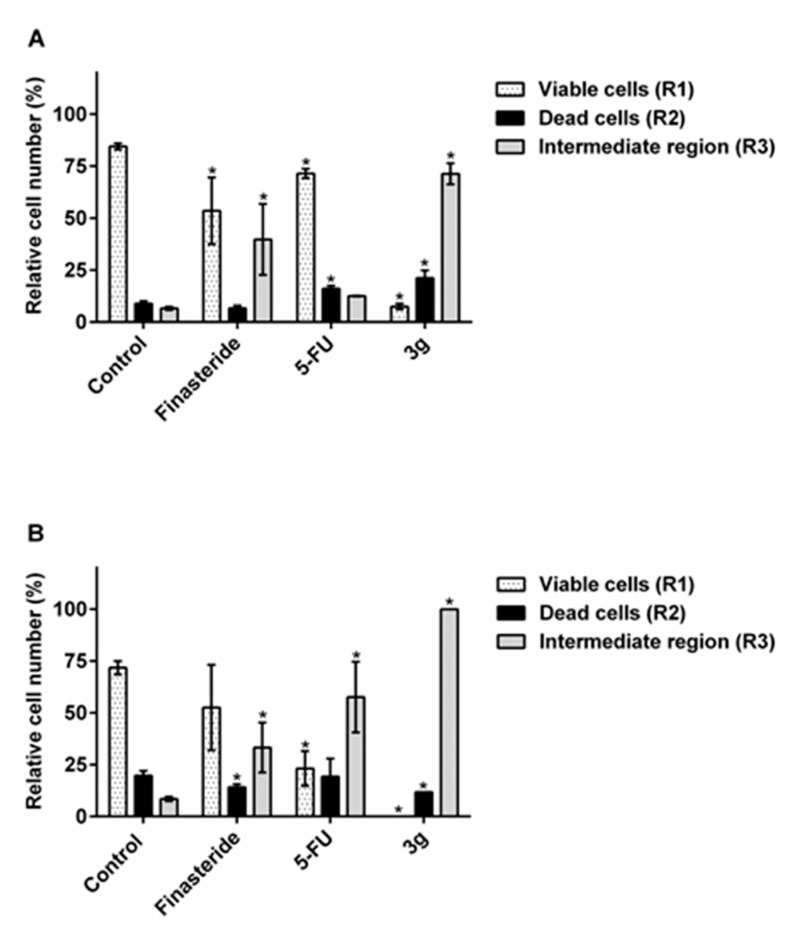
Distribution of the events in the regions R1, R2 and R3 (relative cell number): (**A**) after 24 and (**B**) 72 h of treatment with finasteride, 5-fluorouracil (5-FU) and **3g**. Data are expressed as mean ± SD of the percentage of cells in the different regions (n = 4–6) and are representative of at least two independent experiments. * *p* < 0.05 vs. the control (ANOVA).

Cell viability of LNCaP cells when exposed to steroid **3g**, finasteride and 5-FU was more accurately evaluated by flow cytometry quantification of PI permeant (dead) vs. non-permeant cells (live cells) (Figure 7) [35]. Derivative **3g** induced a drastic reduction in the number of living cells after 24 h of incubation. After 72 h, the number of viable cells was almost absent. On the other hand, it can be noted that after 24 and 72 h of incubation a relevant proportion of cells is in R3, a region that, despite being less well defined, can be associated with partial PI permeability or increased autofluorescence (early apoptotic cells) and/or cell debris with degraded DNA (advanced cell death stage) [35]. This study suggests that the mechanism of cell death by **3g** is different in relation to finasteride or 5-FU. 

### 2.3. Molecular Docking

An in silico study was performed using molecular docking simulations. The principal aim of this study was to assess the affinity and the existence of potential interactions between these new arylidenes and several proteins that are known targets of steroidal drugs currently used in the treatment of BHP, PCa and breast cancer: 5AR type 2, AR, ERα and CYP17A1. The structures of these targets were chosen based on several important criteria, specifically: the existence of high-resolution X-ray crystal structure, available structure in a complex with a steroidal drug or other similar ligands, and, finally, the protein have to be a target of clinically-approved steroid-based anticancer drugs in the treatment of breast or prostate cancers. On this set, 5AR type 2, an important target of 4-azasteroids used in the symptomatic treatment of BPH, is the only exception. Therefore, this study was performed for each 4-azapregnene **3a**–**g** against the mentioned proteins. Three-dimensional structural coordinates of protein receptors were retrieved from the protein data bank (PDB), and molecular docking simulations were executed using AutoDockTools. To validate the docking method, simulations were carried out between crystallized ligands/drugs with the respective proteins and all control re-docking simulations were able to reproduce the ligand–protein interaction geometries presented in the respective crystal structures with a root-mean-square distance (RMSD) ≤ 2.0 Å. The results of re-docking in all simulations exhibit a RMSD equal to or lower than 1.0 Å (Table 3). Based on the control docking simulations, predicted binding energies < −11.00 kcal.mol^−1^ were considered to be significant in the cases of 5AR type 2, AR and CYP17A1, and <−9.00 kcal.mol^−1^ in the case of ERα. 

It is quite evident that very strong binding energies were predicted for all seven steroids for the active site of 5AR type 2, ERα and CYP17A1, while no significant binding was observed for the majority of these derivatives for the active site of AR (Table 4). These results show the possibility that novel synthesized compounds can potentially interact with the 5AR type 2 and possibly act as 5ARIs, which can be explained by the fact these are 4-azasteroids. In fact, all novel derivatives have similar affinity energy values to the control finasteride. Moreover, in the molecular docking with ERα, all steroids showed higher affinity when compared with 17β-estradiol. Concerning CYP17A1, the affinity energy values obtained to these steroidal derivatives are relatively similar to abiraterone. Although these values are higher than the reference (except for **3d**), a potential interaction between these novel steroids and CYP17A1 cannot be discarded. The principal interactions between the macromolecules and the best-scored derivatives in molecular docking simulations were also analyzed. Through the analysis of principal interactions, it is possible to conclude that the amide group of these derivatives could be essential in establishing polar interactions with the different amino acids of studied proteins. Additionally, the important interactions between proteins (specific amino acids) and crystallized ligands were present in docking simulations for best-scored compounds.

## 3. Materials and Methods

### 3.1. Chemistry 

#### 3.1.1. General Considerations

Reagents and solvents were acquired from standard sources and were purified and/or dried whenever crucial using standard procedures before use. Finasterida Tetrafarma^TM^ 5 mg was purchased from *Tetrafarma – Produtos Farmacêuticos*, Lda, Portugal and the active steroid was extracted from the tablets [36,37]. Finasteride was extracted with high purity, based on the NMR spectra acquired. The reactions were performed under heating and magnetic stirring using Heidolph plates. TLC analysis was carried out using 0.20 mm Al-backed silica-gel plates (Macherey-Nagel 60 F254, Duren, Germany), and after elution, the plates were visualized under UV radiation (254 nm) in a CN-15.LC UV chamber. Then a revelation step with an ethanol/sulfuric acid (95:5) mixture, followed by heating at 120 °C was performed. For the isolation and purification of product **1**, a column chromatography, using silica gel (0.063–0.200 mm or 0.040–0.063 mm) acquired from Merck (New Jersey, USA), was used. The eluents used are indicated as a v/v proportion in the experimental procedure. A Büchi R-215 rotavapor system was used for the evaporation of solvents. Attenuated total reflectance (ATR) IR spectra were collected on a Thermoscientific Nicolet iS10: smart iTR, equipped with a diamond ATR crystal. For IR data acquisition, each solid sample was placed onto the crystal and the spectrum was recorded. An air spectrum was used as a reference in absorbance calculations. The sample spectra were collected at room temperature in the 4000–600 cm^−1^ range by averaging 16 scans at a spectral resolution of 2 cm^−1^. ^1^H- and ^13^C-NMR spectra were recorded on a Bruker Avance 400 MHz spectrometer (^1^H NMR at 400.13 MHz and ^13^C NMR at 100.62 MHz), and were processed with the software TOPSPIN (v. 3.1) (Bruker, Fitchburg, WI, USA). Chloroform (CDCl_3_) was the solvent used in these experiments. Chemical shifts are reported in parts per million (δ) relative to TMS or solvent as an internal standard. Coupling constants (*J* values) are reported in hertz (Hz), and splitting multiplicities are described as s = singlet, d = doublet, t = triplet, combinations of above or m = multiplet. Spectra are available on Supplementary Material (S1). ESI-TOF mass spectrometry was performed by the microanalysis service on a QSTAR XL instrument.

#### 3.1.2. Synthesis 

5-Oxo-A-nor-3,5-secopregnan-3-oic acid (**1**)

To a solution of progesterone (943.41 mg, 3 mmol) dissolved in isopropanol (25 mL) was added a warmed solution of sodium carbonate (510 mg, 4.8 mmol) in water (3 mL). This mixture was carried to reflux, and a heated solution of sodium periodate (5.37 g, 25.1 mmol) and potassium permanganate (70 mg, 0.4 mmol) in water (6 mL) was added dropwise over 1 h. Then, the reflux was maintained for an additional 3 h, and after this period the reaction was cooled to 30 °C. The solids formed were removed by filtration with celite, and washed with water. The combined filtrates were concentrated under reduced pressure to eliminate isopropanol. The aqueous residue was cooled and acidified with the concentrated hydrochloric acid aqueous solution until precipitate formation. The product was extracted with ethyl acetate (3 × 80 mL), washed with brine and dried with anhydrous sodium sulfate. After removal of solvent under reduced pressure, the residue was purified by column chromatography (silica gel, ethyl acetate/petroleum ether 40–60 °C, 1:1) to afford **1** as a white solid (983.30 mg, 89%) [24]; mp 168–169 °C. 

4-Azapregn-4-ene-3,20-dione (**2**)

A mixture of **1** (476.8 mg, 1.4 mmol) and ammonium acetate (647.4 mg, 8.4 mmol) in glacial acetic acid (12 mL) was refluxed for 4 h. At the end of the reaction, the mixture was cooled followed by water addition (100 mL). The product was extracted with DCM (3 × 100 mL). The organic phase was washed with brine and dried with anhydrous sodium sulfate. The solvent was removed under reduced pressure to give **2** as an orange solid (433.3 mg, 98%) [25]; mp 152–155 °C. 

General procedure for the preparation of 4-azapregn-5-ene-3,20-dione derivatives **3a–g** by aldol condensation 

A mixture of ethanolic solution (5 mL) of compound **2** (94.64 mg, 0.3 mmol), aldehyde (0.36 mmol) and aqueous solution of potassium hydroxide (100 μL, 50% *w*/*w*) was stirred for 12–24 h at room temperature. The reaction mixture was worked up by first adding water to induce the precipitation (10 mL), and then filtered and washed with water to obtain **3a**–**g** [15].

21*E*-(Phenylmethylidene)-4-azapregn-5-ene-3,20-dione (**3a**) 

Pallid yellow powder (55.9 mg, 46%); mp 220–223 °C; IR (cm^−1^): 3191, 3060, 2937, 2873, 1693, 1674, 1600, 978, 837. ^1^H NMR (CDCl_3_, 400 MHz) δ: 7.72 (1H, s, -NH), 7.49 (3H, m, H_Ar_+21a-CH), 7.40–7.27 (3H, m, H_Ar_), 6.71 (1H, d, *J* = 16.0 Hz, 21-CH), 4.79 (1H, s, 6-H), 1.02 (3H, s, 19-CH_3_), 0.60 (3H, s, 18-CH_3_). ^13^C NMR (CDCl_3_, 101 MHz) δ: 200.11, 169.61, 141.68, 139.88, 134.74, 130.35, 128.92, 128.28, 126.67, 103.31, 63.51, 61.88, 56.89, 47.93, 44.98, 38.78, 34.26, 31.52, 29.65, 28.39, 24.58, 22.75, 20.98, 18.72, 13.53. HRMS (ESI-TOF) *m/z*: [M + H]^+^ Calcd for C_27_H_34_NO_2_ 404.2584; Found 404.2589.

21*E*-[(4-Methylphenyl)methylidene]-4-azapregn-5-ene-3,20-dione (**3b**) 

Pallid orange solid (92.1 mg, 74%); mp 191–193 °C; IR (cm^−1^): 3192, 3059, 2936, 2871, 1675, 1664, 1597, 990, 837, 809.^1^H NMR (CDCl_3_, 400 MHz) δ: 7.75 (1H, s, -NH), 7.47 (1H, d, *J* = 15.9 Hz, 21a-CH), 7.39 (2H, d, *J* = 7.8 Hz, H_Ar_), 7.13 (2H, d, *J* = 7.7 Hz, H_Ar_), 6.67 (1H, d, *J* = 15.9 Hz, 21-CH), 4.79 (1H, s, 6-H), 2.31 (3H, s, 7’-CH_3_), 1.02 (3H, s, 19-CH_3_), 0.59 (3H, s, 18-CH_3_). ^13^C NMR (CDCl_3_, 101 MHz) δ: 200.16, 169.65, 140.85, 139.87, 131.98, 129.66, 128.30, 125.77, 103.36, 63.51, 61.78, 56.89, 47.94, 44.95, 44.01, 38.76, 34.26, 31.51, 29.65, 28.38, 24.58, 24.36, 22.76, 20.97, 18.72, 13.51. HRMS (ESI-TOF) *m/z*: [M + H]^+^ Calcd for C_28_H_36_NO_2_ 418.2741; Found 418.2735. 

21*E*-[(4-Nitrophenyl)methylidene]-4-azapregn-5-ene-3,20-dione (**3c**) 

Pallid orange solid (89.6 mg, 67%); mp 250–251 °C; IR (cm^−1^): 3193, 3075, 2936, 2868, 1681, 1650, 1592, 1514, 1341, 982, 834, 801. ^1^H NMR (CDCl_3_, 400 MHz) δ: 8.18 (2H, d, *J* = 7.7 Hz, H_Ar_), 7.63 (2H, d, *J* = 7.6 Hz, H_Ar_), 7.52–7.43 (2H, m, -NH+21a-CH), 6.79 (1H, d, *J* = 16.0 Hz, 21-CH), 4.76 (1H, s, 6-H), 1.03 (3H, s, 19-CH_3_), 0.61 (3H, s, 18-CH_3_). ^13^C NMR (CDCl_3_, 101 MHz) δ: 199.83, 169.89, 148.49, 143.48, 138.55, 128.80, 124.18, 123.73, 121.23, 103.03, 62.42, 56.87, 47.99, 45,18, 38.83, 31.77, 31.54, 29.60, 25.94, 24.53, 20.98, 18.73, 14.82, 13.61. HRMS (ESI- TOF) *m/z*: [M + H]^+^ Calcd for C_27_H_33_N_2_O_4_ 449.2435; Found 449.2438. 

21*E*-[(4-Methoxyphenyl)methylidene]-4-azapregn-5-ene-3,20-dione (**3d**)

Yellow solid (70.9 mg, 55%); mp 280–282 °C; IR (cm^−1^): 3188, 3067, 2953, 2871, 2855, 1683, 1651, 1593, 1514, 1108, 986, 833, 805. ^1^H NMR (CDCl_3_, 400 MHz) δ: 7.65 (1H, s, -NH), 7.52–7.39 (3H, m, H_Ar_+21a-CH), 6.84 (2H, d, *J* = 8.5 Hz, H_Ar_), 6.60 (1H, d, *J* = 15.9 Hz, 21-CH), 4.78 (1H, s, 6-H), 3.78 (3H, s, 7’-CH_3_), 1.02 (3H, s, 19-CH_3_), 0.59 (3H, s, 18-CH_3_). ^13^C NMR (CDCl_3_, 101 MHz) δ: 200.04, 169.57, 161.49, 141.46, 139.89, 129.98, 127.41, 124.53, 114.38, 103.29, 61.77, 56.89, 55.41, 47.95, 44.93, 44.01, 38.76, 34.28, 31.52, 29.65, 28.40, 24.58, 22.78, 20.94, 18.72, 13.50. HRMS (ESI-TOF) *m/z*: [M + H]^+^ Calcd for C_28_H_36_NO_3_ 434.2690; Found 434.2692. 

21*E*-[(Furan-2-yl)methylidene]-4-azapregn-5-ene-3,20-dione (**3e**) 

Pallid yellow solid (73.0 mg, 62%); mp 260–261 °C; IR (cm^−1^): 3193, 3122, 2937, 2870, 1684, 1662, 1603, 1551, 1014, 974, 815. ^1^H NMR (CDCl_3_, 400 MHz) δ: 7.61 (1H, s, -NH), 7.42 (1H, s, H_Ar_), 7.26 (1H, d, *J* = 15.6 Hz, 21a-CH), 6.60 (2H, d, *J* = 15.6 Hz, H_Ar_+21-CH), 6.42 (1H, dd, *J* = 3.2, 1.8 Hz, H_Ar_), 4.77 (1H, s, 6-H), 1.02 (3H, s, 19-CH_3_), 0.59 (3H, s, 18-CH_3_). ^13^C NMR (CDCl_3_, 101 MHz) δ: 199.74, 169.45, 151.38, 144.63, 139.95, 127.76, 123.90, 115.75, 112.52, 103.15, 62.31, 56.84, 47.92, 44.96, 38.69, 34.28, 31.77, 31.54, 29.64, 28.44, 24.55, 22.67, 20.95, 18.73, 13.48. HRMS (ESI-TOF) *m/z*: [M + H]^+^ Calcd for C_25_H_32_NO_3_ 394.2377; Found 394.2382. 

21*E*-[(5-Methylfuran-2-yl)methylidene]-4-azapregn-5-ene-3,20-dione (**3f**) 

Orange solid (102.6 mg, 84%); mp 200–203 °C; IR (cm^−1^): 3200, 3119, 2937, 2870, 1665, 1626, 1606, 1569, 1019, 969, 784.^1^H NMR (CDCl_3_, 400 MHz) δ: 7.69 (1H, s, -NH), 7.19 (1H, d, *J* = 15.4, 21a-CH), 6.51 (2H, d, *J* = 15.7 Hz, H_Ar_+21-CH), 6.03 (1H, s, H_Ar_), 4.79 (1H, s, 6-H), 2.29 (3H, s, 5’-CH_3_), 1.02 (3H, s, 19-CH_3_), 0.58 (3H, s, 18-CH_3_). ^13^C NMR (CDCl_3_, 101 MHz) δ: 199.79, 169.50, 155.53, 149.99, 139.95, 127.96, 122.24, 117.67, 109.16, 103.21, 63.51, 62.15, 56.86, 47.96, 44.94, 44.01, 38.69, 34.27, 31.55, 29.66, 28.42, 24.57, 22.75, 20.95, 18.73, 13.99. HRMS (ESI-TOF) *m/z*: [M + H]+ Calcd for C_26_H_34_NO_3_ 408.2533; Found 408.2530. 

21*E*-[(Pyridin-3-yl)methylidene]-4-azapregn-5-ene-3,20-dione (**3g**) 

Beige solid (76.0 mg, 63%); mp 275–277 °C; IR (cm^−1^): 3196, 3089, 2971, 2839, 2864, 1698, 1681, 1647, 1606, 1586, 1477, 998, 811. ^1^H NMR (CDCl_3_, 400 MHz) δ: 8.72 (1H, s, H_Ar_), 8.54 (1H, dd, *J* = 4.9, 1.6 Hz, H_Ar_), 7.79 (1H, d, *J* = 7.8 Hz, H_Ar_), 7.71 (1H, s, -NH), 7.47 (1H, d, *J* = 15.9 Hz, 21a-CH), 7.28 (1H, m, H_Ar_), 6.67 (1H, d, *J* = 15.9 Hz, 21-CH), 4.78 (1H, s, 6-H), 1.07 (3H, s, 19-CH_3_), 0.61 (3H, s, 18-CH_3_). ^13^C NMR (CDCl_3_, 101 MHz) δ: 199.60, 169.44, 150.85, 149.71, 139.96, 137.79, 134.60, 130.59, 128.34, 123.79, 103.06, 62.13, 59.31, 56.87, 50.66, 47.88, 45.08, 38.81, 34.27, 31.76, 29.62, 28.43, 24.54, 20.96, 18.73, 13.57. HRMS (ESI-TOF) *m/z*: [M + H]^+^ Calcd for C_26_H_33_N_2_O_2_ 405.2537; Found 405.2534. 

### 3.2. Biology

#### 3.2.1. Cell Culture

Cell lines, namely LNCaP, PC-3, T47-D and NHDF were obtained from American Type Culture Collection (ATCC; Manassas, VA, USA) and cultured in 75 cm^2^ culture flasks at 37 °C in a humidified air incubator with 5% CO_2_. LNCaP, PC-3 and T47-D cells, which were used in passages 20th to 27th, 25th to 29th and 10th to 15th, respectively, were cultured in RPMI 1640 medium (Sigma-Aldrich, Inc., St. Louis, MO, USA) with 10% fetal bovine serum (FBS; Sigma-Aldrich, Inc. St. Louis, MO, USA) and 1% of the antibiotic mixture of 10,000 IU/mL penicillin G and 100 mg/mL of streptomycin (Sp, Sigma-Aldrich, Inc. St. Louis, MO, USA). Finally, NHDF cells (Normal Human Dermal Fibroblasts) were cultured in RPMI 1640 medium supplemented with 10% FBS, 2 mM *L*-glutamine, 10 mM HEPES, 1 mM sodium pyruvate and 1% of an antibiotic/antimycotic mixture (10,000 U/mL penicillin G, 100 mg/mL streptomycin and 25 µg/mL amphotericin B) (Ab; Sigma-Aldrich, Inc. St. Louis, MO, USA), and these cells were used in passages 10 to 12. For all cell types, the medium was renewed every 2–3 days until cells reach nearly the confluence state. Then, they were detached gently by trypsinization (trypsin-EDTA solution: 0.125 g/L of trypsin and 0.02 g/L of EDTA). 

#### 3.2.2. Preparation of Compounds Solutions

All the stock solutions were prepared by dissolving compounds in dimethyl sulfoxide (DMSO; Sigma-Aldrich, Inc., St. Louis, MO, USA) at 10 mM and stored at 4 °C. From these solutions, the several diluted compounds solutions in different concentrations were freshly prepared in a complete culture medium before each experiment. The DMSO concentration in final solutions did not interfere with the cell viability (<1%). 

#### 3.2.3. MTT Cell Proliferation Assay [38]

As previously referred, cells were trypsinized after reaching a near confluence state and counted by the trypan blue exclusion assay in a Neubauer chamber. Then, 100 µL of cell suspension/well with an initial density of 2 × 10^4^ cells/mL were seeded in 96-well culture plates (Nunc, Apogent, Denmark) and left to adhere for 48 h. After cells adherence, the medium was replaced by the distinct solutions of the compounds in the study (30 µM for preliminary studies and 0.01, 0.1, 1, 10, 50 and 100 µM for concentration–response studies) in the appropriate medium for approximately 72 h. 5-FU and finasteride were used as positive controls and untreated cells were used as the negative control. Each experiment was performed in quadruplicate and independently performed at least two times. The in vitro antiproliferative effects were evaluated by the MTT assay (Sigma-Aldrich, Inc. St. Louis, USA). After the incubation period, the medium was removed and 100 µL of phosphate buffer saline (NaCl 137 mM, KCl 2.7 mM, Na_2_HPO_4_ 10 mM and KH_2_PO_4_ 1.8 mM in deionized water and pH adjusted to 7.4) were used to wash the cells. Then, 100 µL of the MTT solution (5 mg/mL) was prepared in the appropriate serum-free medium and was added to each well, followed by incubation for 4 h at 37 °C. Hereafter, the MTT containing medium was removed and the formazan crystals were dissolved in DMSO. Then the absorbance was measured at 570 nm using a microplate spectrophotometer BIO-RAD xMarkTM. Cell viability values were expressed as percentages relative to the absorbance determined in the cells used as negative controls.

#### 3.2.4. Fluorescence Microscopy Assay

To access the effects of the compound **3g** in T47-D cells, fluorescence microscopy assays with Hoechst 3342 and PI staining were performed. T47-D cells were seeded at 2 × 10^4^ cells/mL in a 24-well culture plate containing circular coverslips of 10 mm diameter, in a complete culture medium. After 48 h, cells were incubated during 24, 48 and 72 h, with 5-FU at 20 µM, as the positive control, steroid **3g** at 20 µM, and untreated cells were used as a negative control. Then, cells were incubated with 20 µL/per well of a solution of PI in PBS at 1 mg/mL for 25 min at 37 °C. The medium was removed, and the cells were fixed with formalin 4% for 15 min at room temperature. Cells were washed three times with PBS and then they were incubated with 30 µL/per coverslip of Hoechst 33,342 (1:500) for 10 min. After incubation, cells were washed three times with PBS. Coverslips were mounted on a drop of permanent mounting medium (Dako) on a microscope slide and visualized in a Zeiss Axio Imager A1 microscope with the 40 × objective. The PI/Hoechst 3342 ratio was calculated by dividing PI-positive cells by Hoechst 3342-positive cells and, then the values were normalized.

#### 3.2.5. Cell Nuclear Morphology and Distribution Analysis with Imagej

The nuclear measurements were achieved by converting 16-bit photomicrographs of Hoechst 3342-stained nuclei, in different conditions, into 8-bit images and then these images were autothresholded to binary photos using the default method “Make binary” function of ImageJ v 1.49. Cell nuclei that are touching were separated and fragments were discarded based on the area through the “Analyze Particle” function. This function also provides several information pretended as nuclear area, circumference and form factor [33]. In addition, the “Nearest Neighbor Distance” was determined. This function allows measuring the distance between each cell nucleus and the nearest ones.

#### 3.2.6. Flow Cytometry Assay

Similar to the previous study, the analysis of cell viability was accomplished by flow cytometry after staining dead cells with propidium iodide (PI; Invitrogen, Carlsbad, USA) [20]. Succinctly, 1 mL of a cell suspension was seeded in 12-well culture plates (initial cell density of 5 × 10^4^ cells/mL of LNCaP cells for 24 h assay and 2 × 10^4^ cells/mL of the same cell line for 72 h assay) in a complete culture medium. After 48 h, the cells were treated with finasteride and 5-FU, as positive controls, and compound **3g** at a concentration of 50 µM. Untreated cells were used as a negative control. At the pretended time point, the supernatant was collected and pooled with the cells harvested by trypsin treatment (each well was also washed with 400 µL of PBS before trypsin treatment). The resulting cell suspension was kept on ice and pelleted by centrifugation and resuspended with 400 µL of complete medium. Afterward, 5 µL of PI (1 mg/mL) was transferred to a FACS tube and 395 µL of the cell suspension was added to this tube. A total of 20,000 events (very small events excluded) were acquired using a FACSCalibur flow cytometer, using the FCS, SSC and FL3 (PI) channels. Acquisition and analysis were executed with CellQuestTM Pro (v. 5.1) software. Briefly, a region was created on the SSC/FCS contour plot to exclude events of very small size and complexity (considered not relevant debris). Then, in the FCS/FL3 contour plot gated on the previous region, three regions were created, one corresponding to viable cells, R1, another to dead cells, R2, and another corresponding to an intermediate subpopulation of cells, R3, which may include both large debris resulting from apoptotic death (with very low DNA content) and cells with partial permeability to PI (plots available on Supplementary material, S2). The percentages of each region were calculated for a total of R1 + R2 + R3. The experiment was performed in two dependent days, each in duplicate or triplicate wells.

#### 3.2.7. Caspase-9 Activity Assay 

Promega Caspase-Glo 9 assay (Promega, Madison, WI, USA) was employed to evaluate the caspase-9 activity. T47-D cells were seeded in a 96 multiwell plate at a density of 4 × 10^4^ cells/mL. After 48 h, cells were treated with the positive control, DOX, at 10 μM and with 10 and 20 μM of **3g**, during 48 h. The assay was performed at the end of treatment, following the instructions provided by the manufacturer. Data are representative of at least two experiments (n = 4 in each). 

#### 3.2.8. Statistical Data Analysis

The data are expressed as a mean ± standard deviation (SD) and differences between groups were considered statistically significant when 𝑝 < 0.05. The IC_50_ values were determined through sigmoidal fitting analysis with variable slope and considering a 95% confidence level. All data shown are representative of at least two independent experiments.

### 3.3. Molecular Docking 

#### 3.3.1. Preparation of Proteins

The three-dimensional structural coordinates for 5α-reductase type 2 (5AR PDB code: 7BW1), estrogen receptor-α (ERα PDB code: 1A52), androgen receptor (AR PDB code: 2AMA), 17α-hydroxylase-17,20-lyase (CYP17A1 PDB code: 3RUK) and aromatase (PDB code: 3EQM) were downloaded from Protein Data Bank (www.rcsb.org, accessed on 12 August 2022). The coordinates of the ligands co-crystallized and water molecules were deleted using the software Chimera (v. 1.10.1), and histidine charges were defined to match the physiologic environment and the final structures were saved in PDB format. Then, non-polar hydrogens were merged in AutoDockTools (v. 1.5.6) from The Scripps Research Institute [39]. Kollman and Gasteiger partial charges were added. Finally, the prepared structures were converted from the PDB format to PDBQT for posterior employment in the docking study. 

#### 3.3.2. Preparation of Ligands

Chem3D (v. 12.0) software (by Cambridge ChemBioOffice 2010) was used to build ligands. Then, geometry optimization and energy minimization (MMFF94 force field: 500 steps of conjugate gradient energy minimization followed by 500 steps of steepest descent energy minimization with a convergence setting of 10 × 10^−7^) were executed with Avogadro (v. 1.0.1). The final structures were saved in a PDB file format. The ligands were completely prepared, choosing torsions and the structures were converted from PDB format to PDBQT, in AutoDockTools software.

#### 3.3.3. Grid Map Calculations

AutoGrid4 was used to calculate Autodock grid maps for each macromolecule, based on the active site coordinates of the crystal structure. The size of all grid boxes was 40 × 40 × 40 with 0.375 Å of spacing. Maps were calculated for each atom type in each ligand along with an electrostatic and desolvation map using a dielectric value of −0.1465.

#### 3.3.4. Molecular Docking Simulations

Molecular docking studies were performed using the Lamarckian genetic algorithm and empirical free energy scoring function [39]. The maximum number of energy evaluations was 2,500,000, and the GA population size was 150. A total of 15 hybrid GA-LS runs were performed for each simulation. The results of these simulations were visualized in PyMol (The PyMol Molecular Graphics System v. 1.3, Schrödinger, LLC – www.pymol.org), built for educational use. All docking simulations conducted to validate the method, using the ligands present in crystal structures, were able to reproduce the ligand–protein interaction geometries. For the docking process to be considered successful, the RMSD value between ligand conformations (docked ligand and crystalized ligand) was less than 2.0 Å.

## 4. Conclusions

A new series of 4-azapregn-5-ene-3,20-diones bearing aromatic or heteroaromatic substituents at C21 were prepared by aldol condensation in good overall yields. The MTT cell proliferation assay showed some interesting cytotoxic activity of some of these compounds in tumor cell lines. In fact, 4-azapregnene derivatives **3a**, **3c** and **3g** presented relevant antiproliferative effects in tumor cells. The pyridinyl derivative, **3g**, had the lowest determined IC_50_ in LNCaP (10.20 µM) and T47-D cells (1.33 µM). The most cytotoxic steroid on PC-3 cells was **3c** (IC_50_ of 3.29 µM), but **3g** also had a very close IC_50_ value (3.64 µM). Therefore, considering these results, steroid **3g** was selected for further biological studies in T47-D cells. The assessment of cytotoxic effect was performed through fluorescence microscopy after PI/Hoechst 3342 staining and caspase-9 activity measurement. All the results indicate that this compound possibly led cells to death by triggering the apoptotic pathway. Moreover, from a flow cytometry assay with PI staining and microscopic observation, a drastic effect on cell viability can be observed when LNCaP cells were exposed to steroid **3g**, which is a very interesting result to explore in a further investigation. Molecular docking studies indicated that these novel 4-azapregnene derivatives can potentially interact with 5AR type 2, similarly to finasteride. Additionally, these simulations also showed the possibility of these novel derivatives interacting with the other tested targets of steroidal drugs, with the exception of AR. In conclusion, derivatives **3a**, **3c** and **3g** presented a remarkable cytotoxic effect on tumor cell lines.

## Figures and Tables

**Table 1:** New 21-arylidene-4-azapregn-5-ene derivatives synthesized, and respective overall yields (%) ^1^.

Aldehyde	Arylidenesteroid	Overall Yield (%)
Benzaldehyde	**3a**	40
*p*-tolualdehyde	**3b**	65
4-nitrobenzaldehyde	**3c**	58
4-methoxybenzaldehyde	**3d**	48
2-furaldehyde	**3e**	54
5-methyl-2-furaldehyde	**3f**	73
Pyridine-3-carboxaldehyde	**3g**	55

^1^ The overall yields are the global yield after the three synthetic steps from progesterone.

**Table 2:** In vitro antiproliferative activities of the tested compounds against LNCaP, PC-3, T47-D and NHDF cell lines, after 72 h of exposure. MTT screening results at 30 µM (data presented as average percentage of negative control ± SD) and determined IC_50_ values (µM) with the respective R squared (R^2^) ^1^.

Compound		LNCaP	PC-3	T47-D	NHDF
**Progesterone**	Screening (30 µM)	64.3 ± 16.1	42.9 ± 5.4	62.9 ± 6.8	96.6 ± 14.3
	IC_50_ (µM); R^2^	--	--	--	--
**Testosterone**	Screening (30 µM)	99.2 ± 4.2	89.5 ± 9.0	72.0 ± 4.0	94.2 ± 7.3
	IC_50_ (µM); R^2^	--	--	--	--
**DHT**	Screening (30 µM)	138.9 ± 3.9	93.1 ± 4.9	90.7 ± 9.4	100.5 ± 7.4
	IC_50_ (µM); R^2^	--	--	--	--
**1**	Screening (30 µM)	96.4 ± 16.1	102.1 ± 5.2	130.8 ± 20.8	100.0 ± 6.0
	IC_50_ (µM); R^2^	--	--	--	--
**2**	Screening (30 µM)	58.3 ± 13.6	96.7 ± 9.3	137.0 ± 18.0	106.1 ± 6.0
	IC_50_ (µM); R^2^	--	--	--	--
**3a**	Screening (30 µM)	6.5 ± 5.3	1.4 ± 1.0	6.5 ± 5.4	25.1 ± 7.4
	IC_50_ (µM); R^2^	27.47; 0.93	12.60; 0.96	16.94; 0.97	63.99; 0.91
**3b**	Screening (30 µM)	62.7 ± 9.0	25.9 ± 3.8	45.0 ± 11.6	83.0 ± 3.6
	IC_50_ (µM); R^2^	--	25.94; 0.98	44.99; 0.98	--
**3c**	Screening (30 µM)	5.2 ± 2.6	0.7 ± 0.5	6.7 ± 4.6	20.3 ± 2.8
	IC_50_ (µM); R^2^	16.26; 0.97	3.29; 0.99	6.56; 0.98	6.94; 0.93
**3d**	Screening (30 µM)	88.0 ± 15.5	58.2 ± 6.0	73.9 ± 14.6	52.4 ± 2.8
	IC_50_ (µM); R^2^	--	--	--	--
**3e**	Screening (30 µM)	85.3 ± 6.7	82.7 ± 6.5	92.6 ± 7.4	111.6 ± 13.9
	IC_50_ (µM); R^2^	--	--	--	--
**3f**	Screening (30 µM)	88.9 ± 17.7	108.1 ± 7.8	104.3 ± 11.3	89.7 ± 9.7
	IC_50_ (µM); R^2^	--	--	--	--
**3g**	Screening (30 µM)	0.7 ± 1.0	0.1 ± 0.1	1.0 ± 0.4	23.8 ± 3.2
	IC_50_ (µM)	10.20; 0.90	3.64; 0.98	1.33; 0.98	3.09; 0.90
**Finasteride**	Screening (30 µM)	88.5 ± 2.4	78.8 ± 8.7	103.5 ± 17.8	62.8 ± 5.9
	IC_50_ (µM); R^2^	--	--	--	--
**5-FU**	Screening (30 µM)	18.6 ± 2.6	10.8 ± 3.1	42.3 ± 6.4	33.6 ± 8.2
	IC_50_ (µM); R^2^	1.50; 0.98	3.30; 0.99	0.70; 0.99	29.44; 0.97

^1^ The cells were treated with the following distinct concentrations: 0.01, 0.1, 1, 10, 50 and 100 µM for 72 h. The antiproliferative effect was accessed by the MTT assay and the IC_50_ values were determined by sigmoidal fitting. Data shown are representative of at least two independent experiments. DHT = 5α-dihydrotestosterone; 5-FU = 5-fluorouracil.

**Table 3:** Results of re-docking using GA of protein–ligand complexes, PDB accession codes and resolution ^1^.

Protein–Ligand Complex	PDB Code	Resolution (Å)	Number of Clusters	Energy (kcal.mol^−1^) and RMSD (Å)			
				Lowest Energy	RMSD of Lowest Energy	Average Energy	Average RMSD
**5AR type 2 +** **Adduct of Finasteride/NADPH**	7BW1	2.8	1	−11.90	0.78	−6.89	1.80
**ERα + 17β-estradiol**	1A52	2.8	1	−9.28	0.51	−9.28	0.53
**AR + DHT**	2AMA	1.9	1	−11.20	0.79	−11.20	0.79
**CYP17A1 + Abiraterone**	3RUK	2.6	1	−11.78	0.23	−11.78	0.29

^1^ 5AR = 5α-reductase; NADPH = nicotinamide adenine dinucleotide phosphate; Erα = estrogen receptor α; AR = androgen receptor; DHT = 5α-dihydrotestosterone; CYP17A1 = cytochrome P450 family 17 subfamily A member 1.

**Table 4:** Predicted binding energies of steroids **3a**–**g** calculated from molecular docking against known protein targets of steroidal molecules: 5AR type 2, ERα, AR and CYP17A1. Binding energies of ligands (in bold) present in the X-ray crystal structures were calculated by re-docking ^1^.

Compound	5AR Type 2	ERα	AR	CYP17A1
*Autodock binding energies (kcal.mol^−1^)*				
**3a**	−11.79	−10.87	−9.38	−10.24
**3b**	−12.02	−11.44	−8.88	−11.17
**3c**	−11.70	−10.43	−5.10	−9.32
**3d**	−11.63	−11.05	−7.32	−11.99
**3e**	−11.07	−10.78	−9.70	−10.91
**3f**	−11.24	−11.43	−9.98	−10.95
**3g**	−11.71	−10.67	−10.35	−10.93
**Finasteride**	**−11.90**	−	−	−
**17β-estradiol**	−	**−9.28**	−	−
**DHT**	−	−	**−11.20**	−
**Abiraterone**	−	−	−	**−11.78**

^1^ 5AR = 5α-reductase; Erα = estrogen receptor α; AR = androgen receptor; CYP17A1 = cytochrome P450 family 17 subfamily A member 1; DHT = 5α-dihydrotestosterone.

## Data Availability

Not applicable.

## Supplementary Materials

The following supporting information can be downloaded at: www.mdpi.com/article/10.3390/molecules27186126/s1. NMR and IR spectra of synthesized compounds (S1). Counter plots obtained from flow cytometry after PI staining are also available for control, finasteride and steroid **3g** (S2).

## Sample Availability

Samples of the compounds are not available from the authors.
