# Supplementary material for: Novel 4-Azapregnene Derivatives as Potential Anticancer Agents: Synthesis, Antiproliferative Activity and Molecular Docking Studies
Source: Molecules. 2022 Sep 19;27(18):6126. doi: 10.3390/molecules27186126 (PMC9501232; doi:10.3390/molecules27186126)
Supplement: Supplementary file 1 [file molecules-27-06126-s001.zip › molecules-1908933-SI.pdf]

Supplementary Material

Novel 4-Azapregnene Derivatives as Potential Anticancer Agents: Synthesis, Antiproliferative Activity and Molecular Docking Studies

Vanessa Brito [1], Adriana Oliveira Santos [1], Gilberto Alves [1], Paulo Almeida [1] and Samuel Silvestre [1,2,*]

[1] Health Sciences Research Centre (CICS-UBI), Universidade da Beira Interior, Av. Infante D. Henrique, 6200-506, Covilhã, Portugal; vanessa.sofia.brito@ubi.pt (V.B.); aosantos@fcsaude.ubi.pt (A.O.S.); gilberto@fcsaude.ubi.pt (G.A.); pjsa@ubi.pt (P.A.)

[2] Center for Neuroscience and Cell Biology (CNC), University of Coimbra, 3004-517, Coimbra, Portugal

* Correspondence: sms@ubi.pt

Table of Contents

Structural characterization of novel compounds	S1
Flow cytometry additional data	S2

^{1}H NMR spectrum of compound 3a

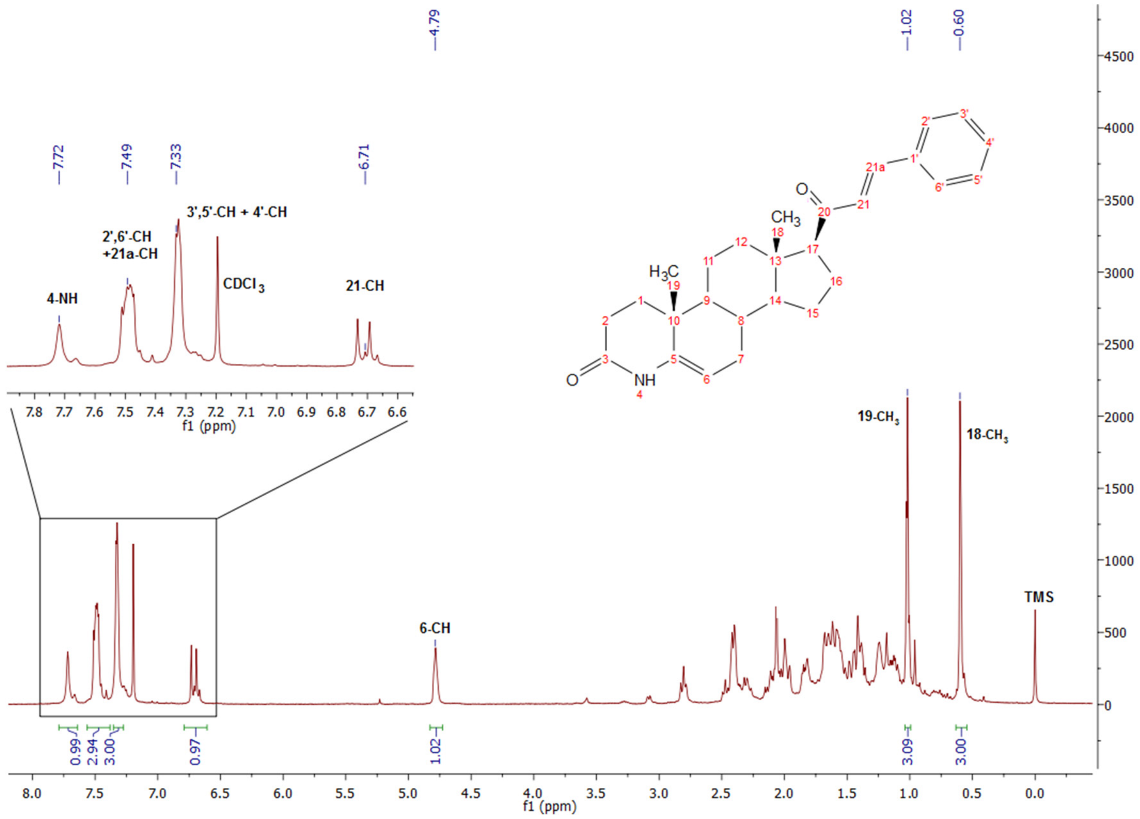

^{13}C NMR spectrum of compound 3a

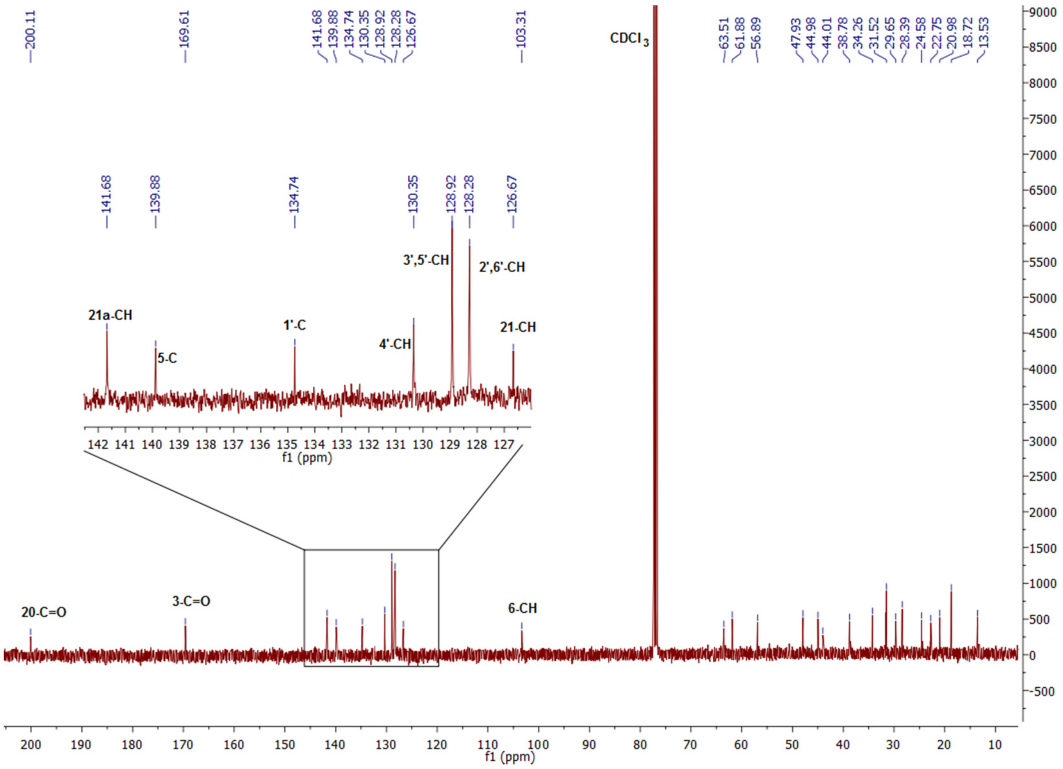

IR spectrum of compound 3a

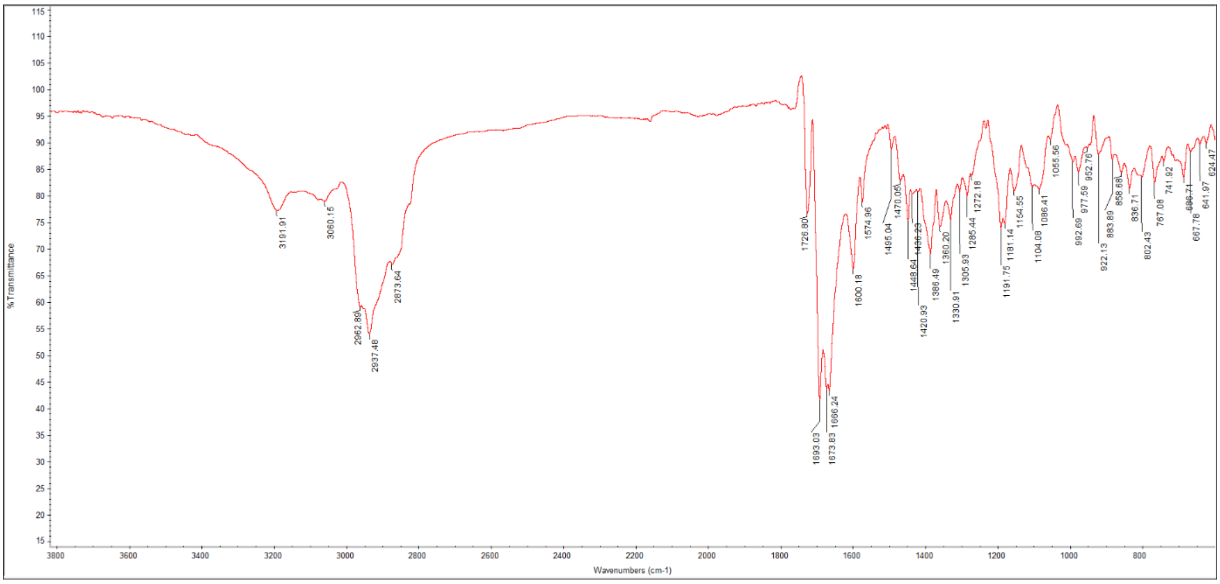

^{1}H NMR spectrum of compound 3b

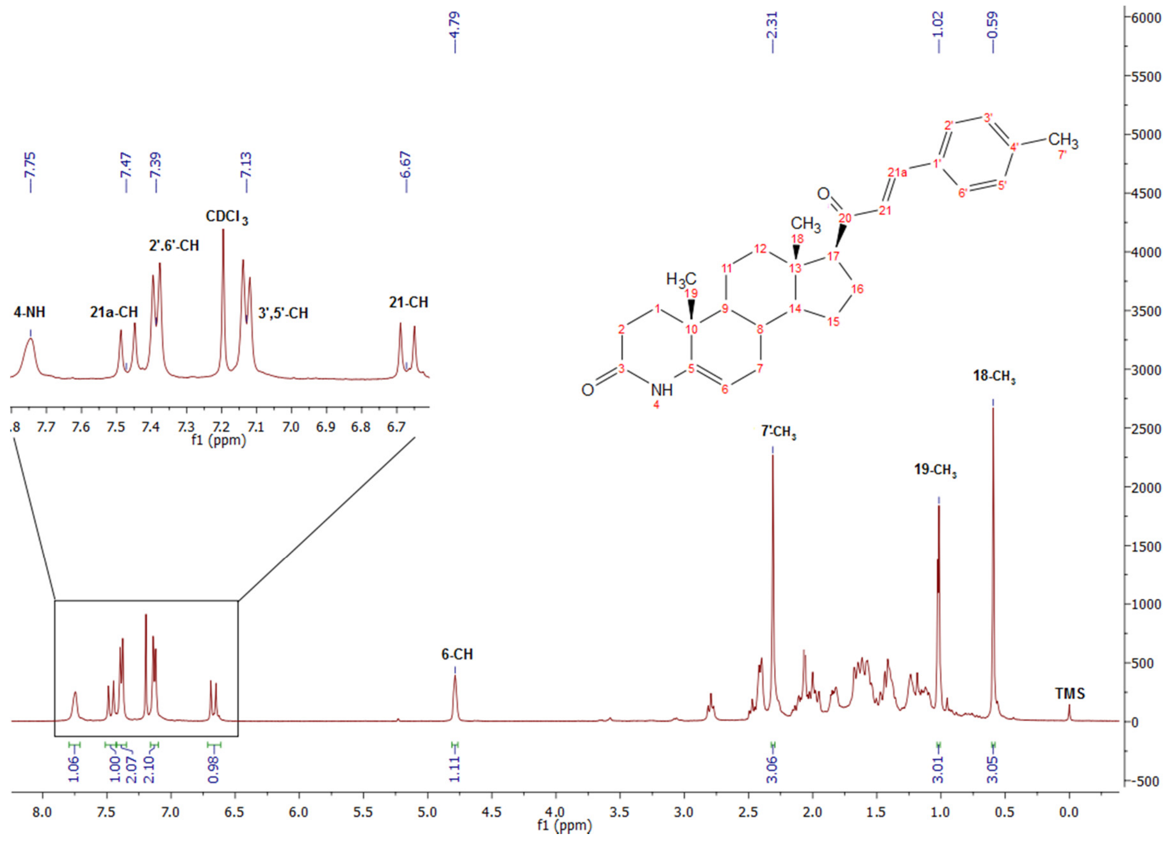

^{13}C NMR spectrum of compound 3b

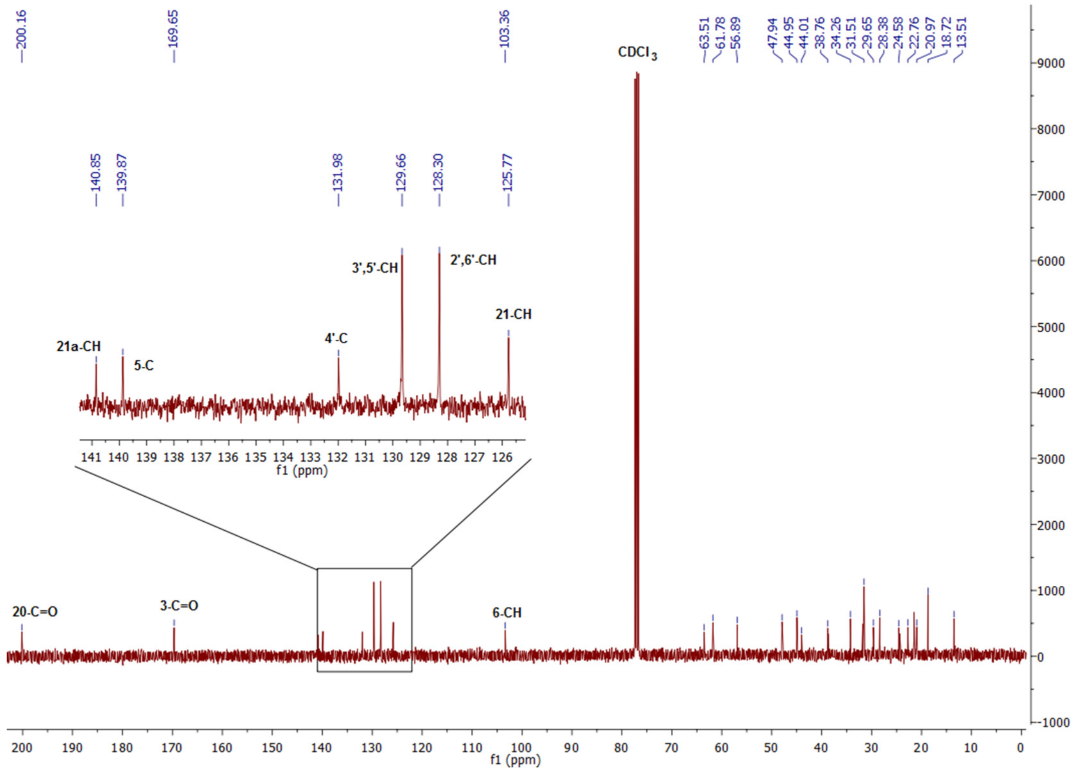

IR spectrum of compound 3b

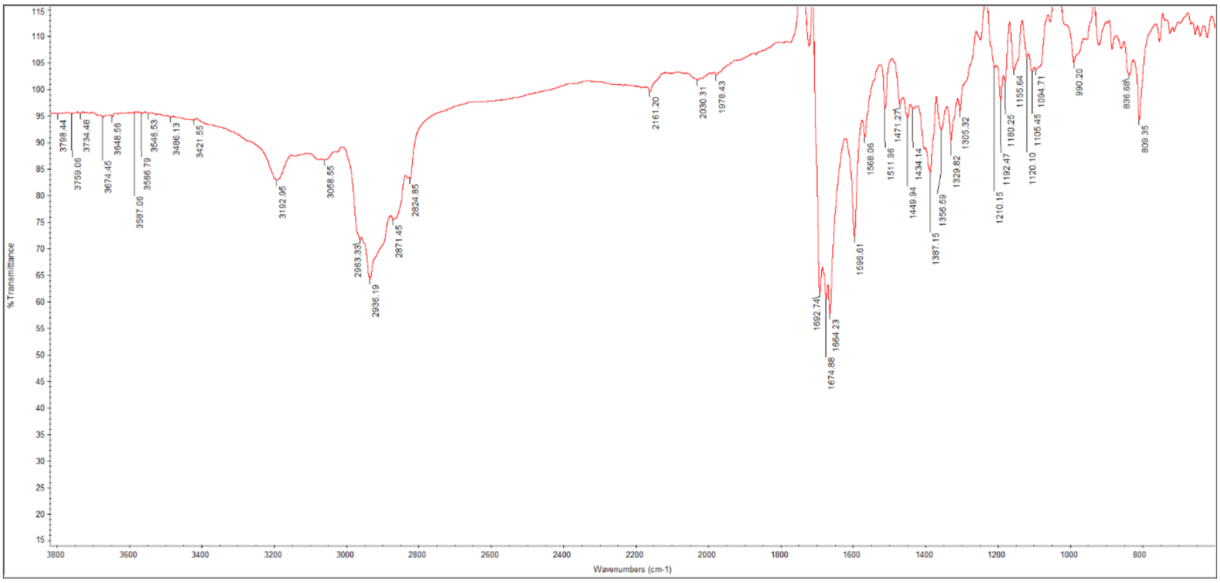

^{1}H NMR spectrum of compound 3c

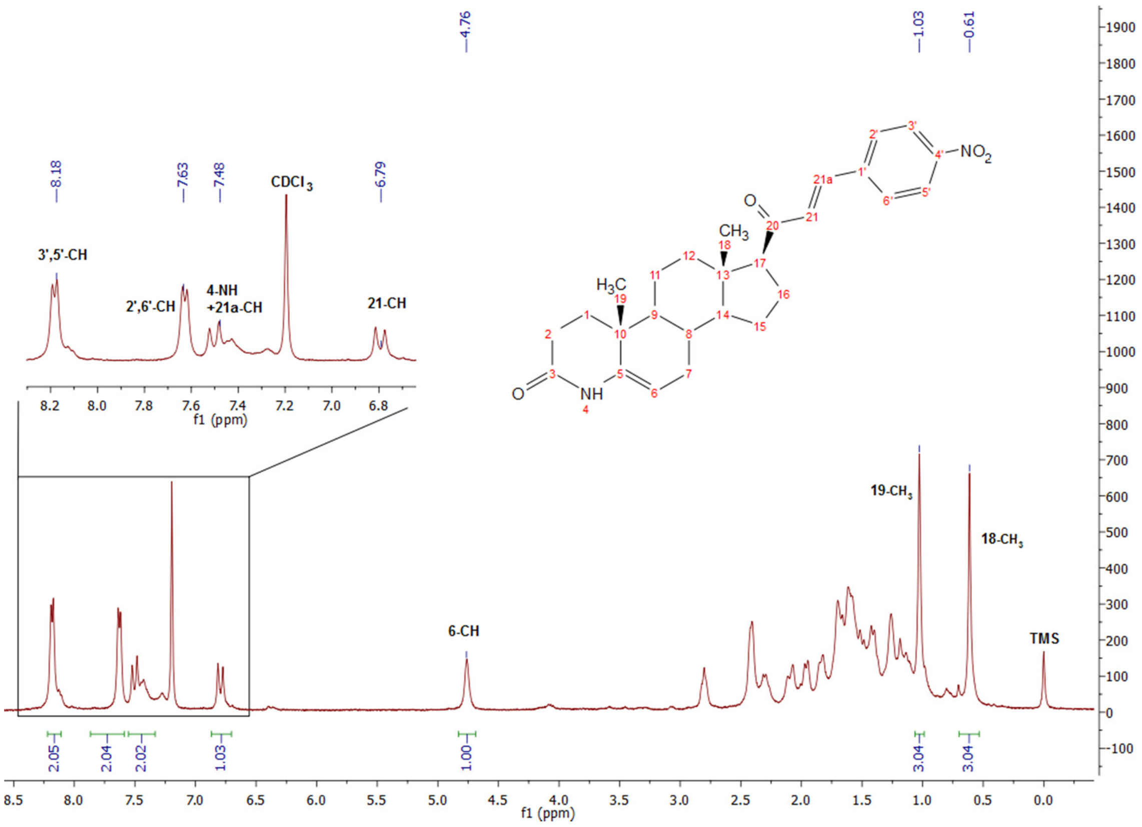

^{13}C NMR spectrum of compound 3c

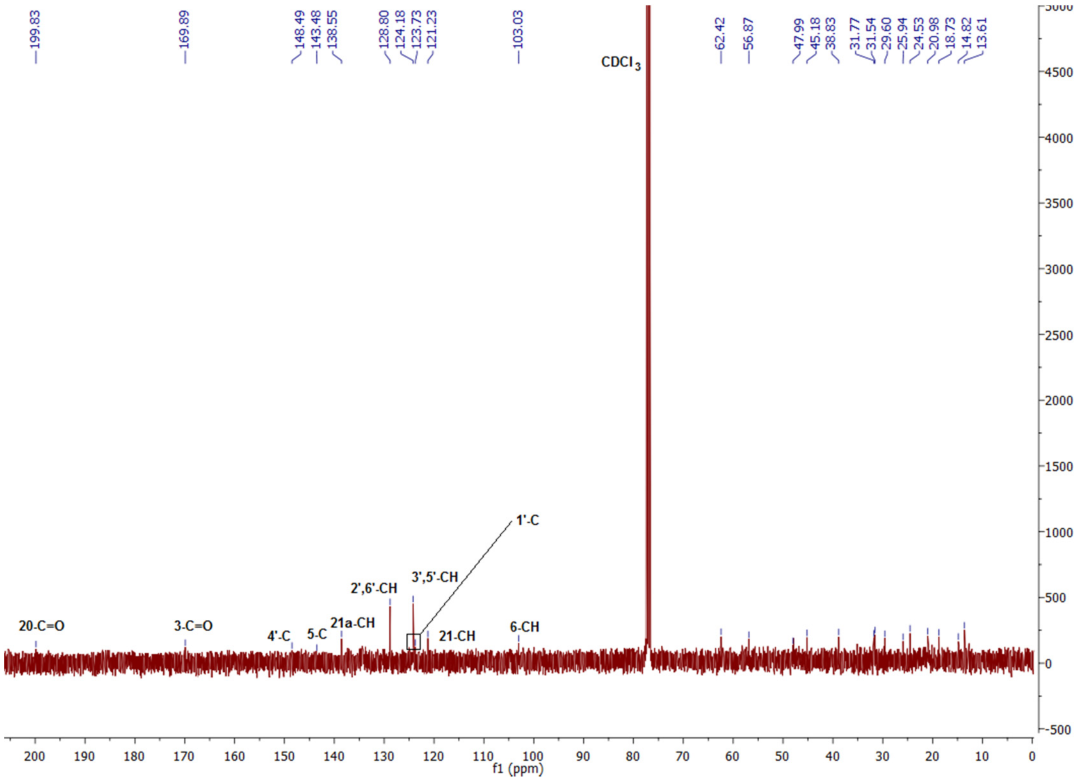

IR spectrum of compound 3c

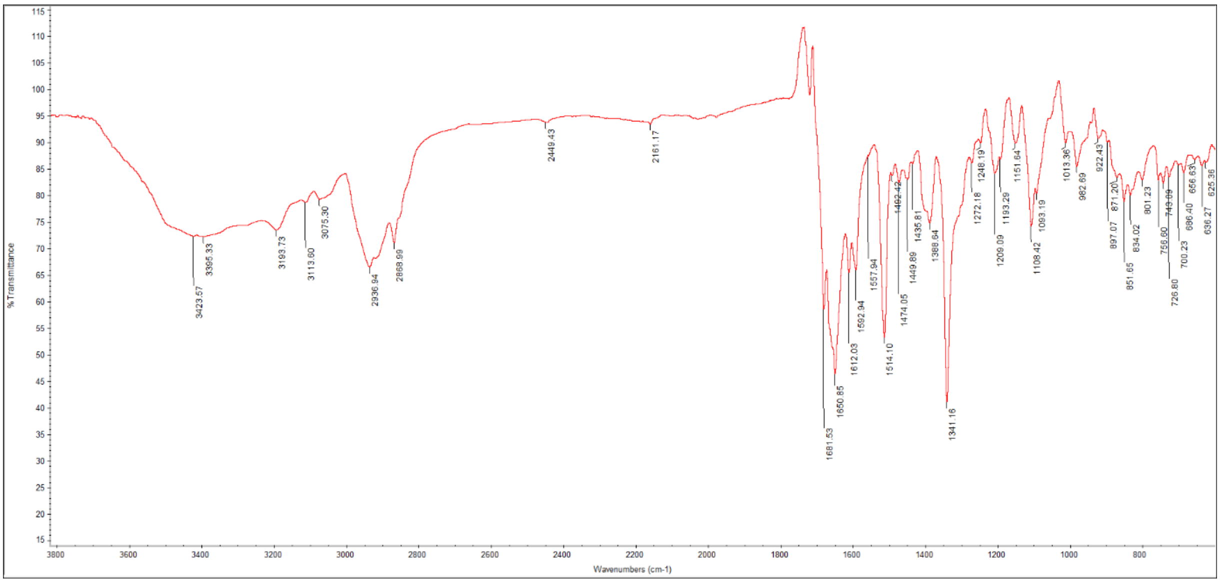

^{1}H NMR spectrum of compound 3d

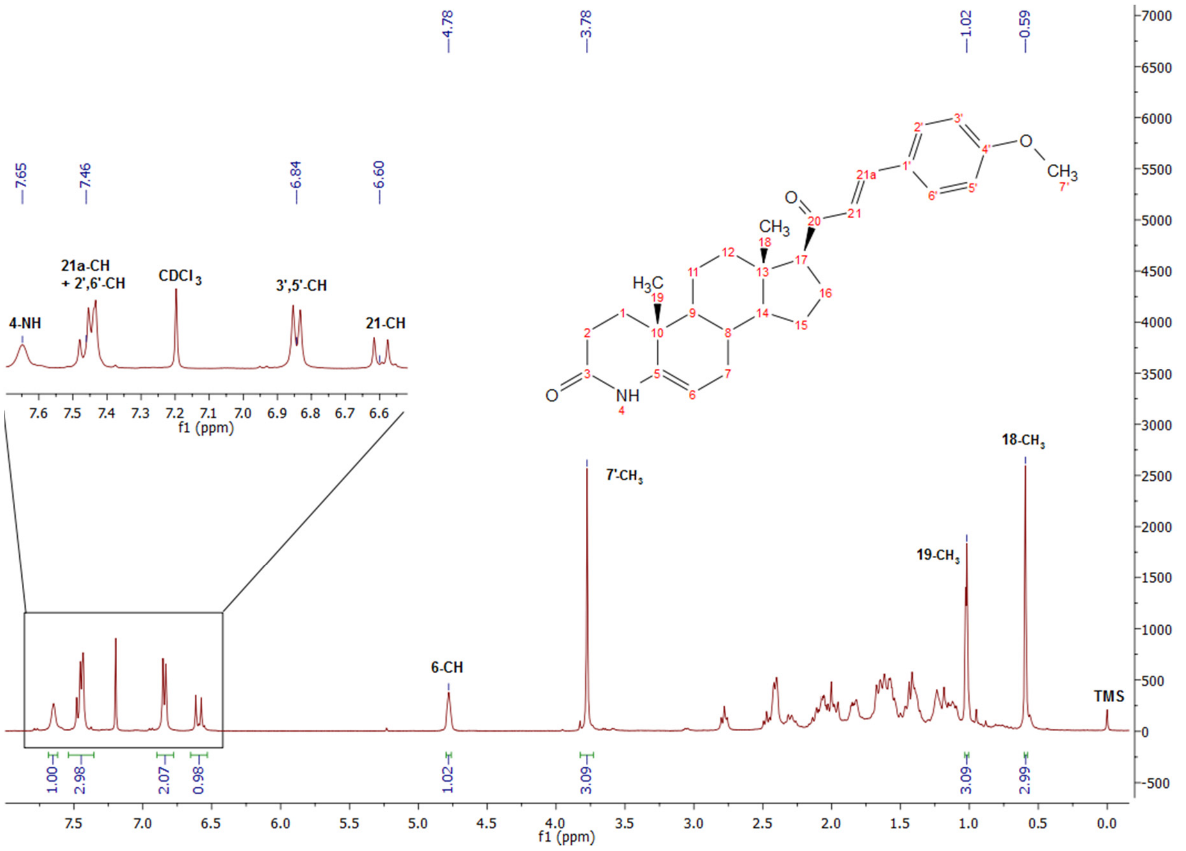

^{13}C NMR spectrum of compound 3d

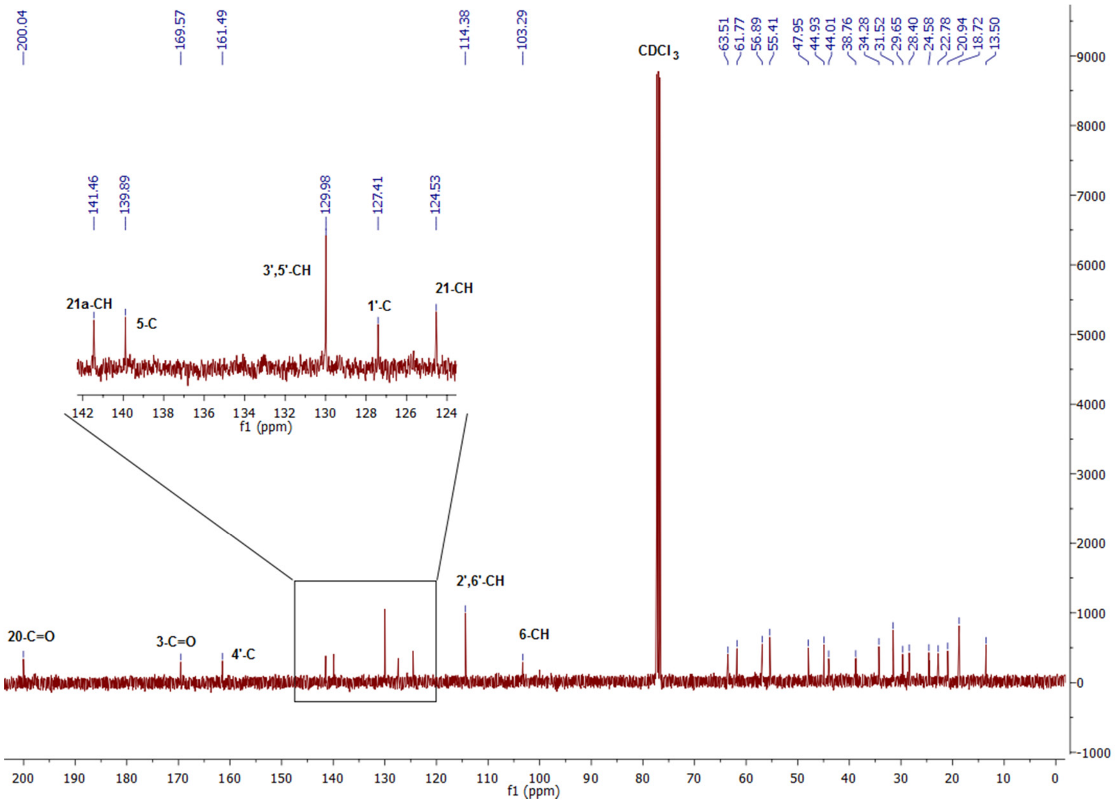

IR spectrum of compound 3d

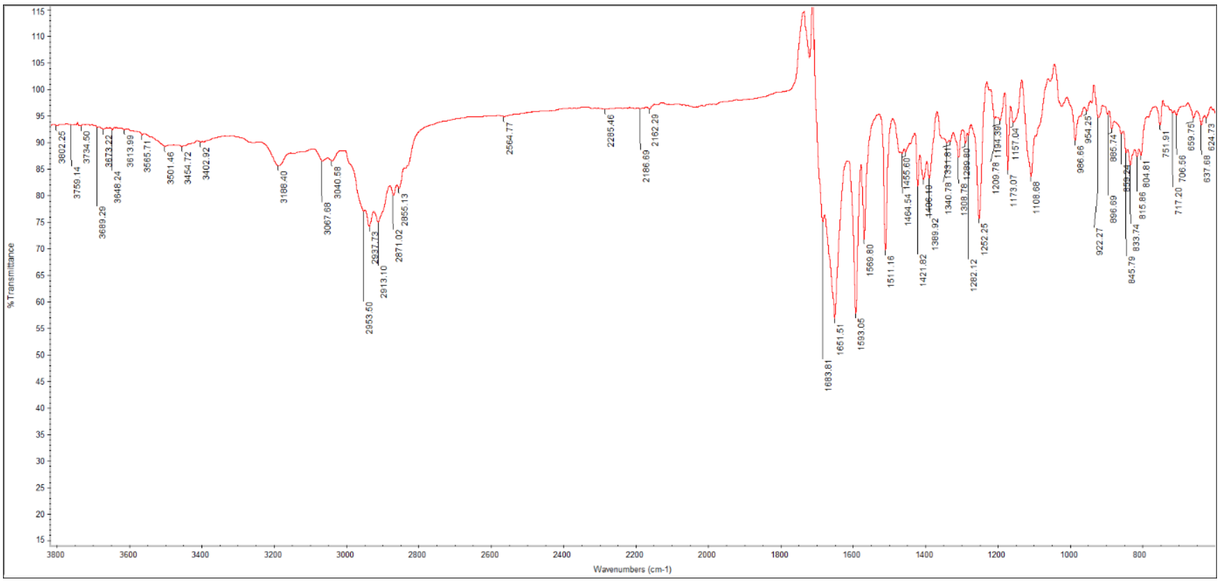

^{1}H NMR spectrum of compound 3e

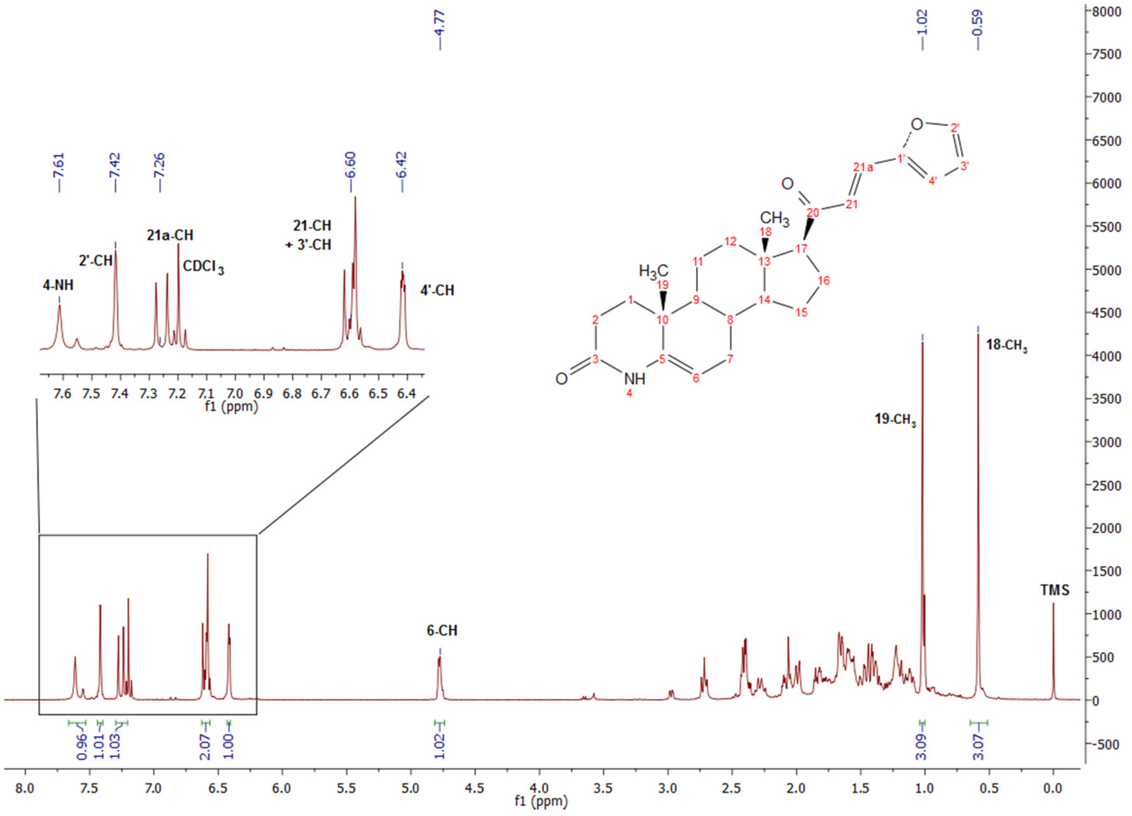

^{13}C NMR spectrum of compound 3e

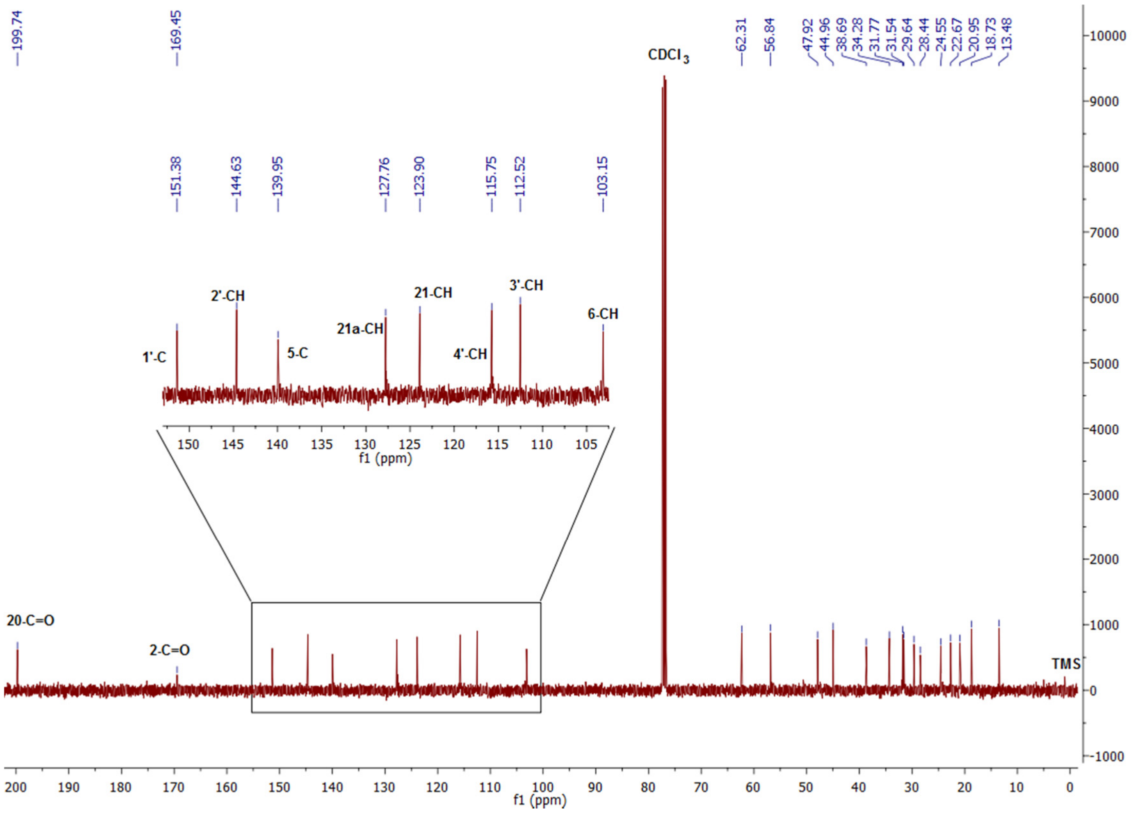

IR spectrum of compound 3e

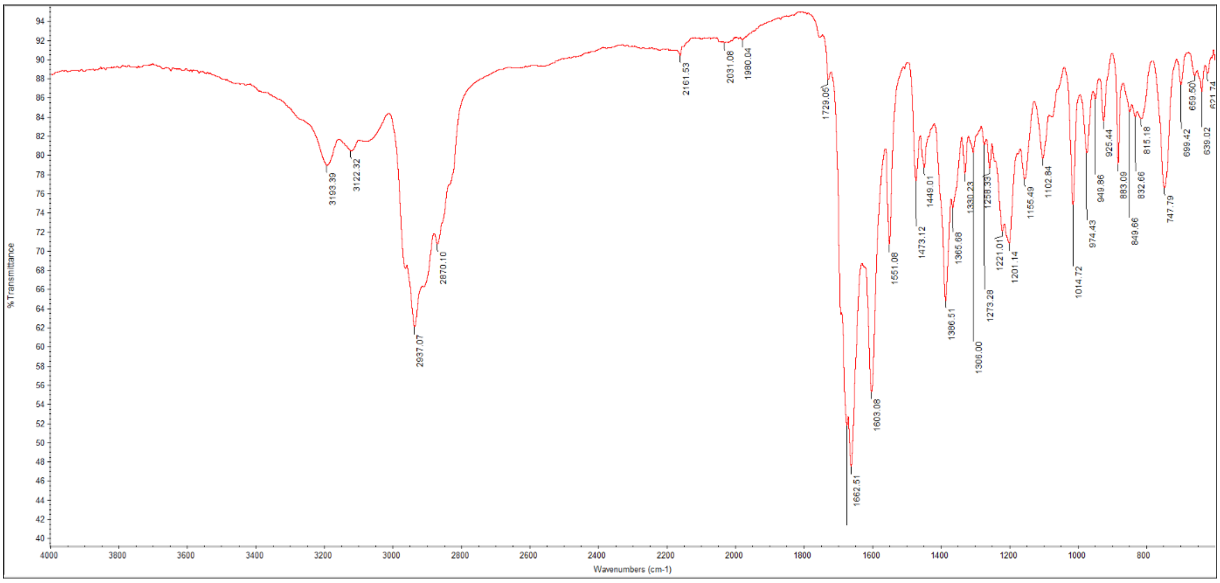

^{1}H NMR spectrum of compound 3f

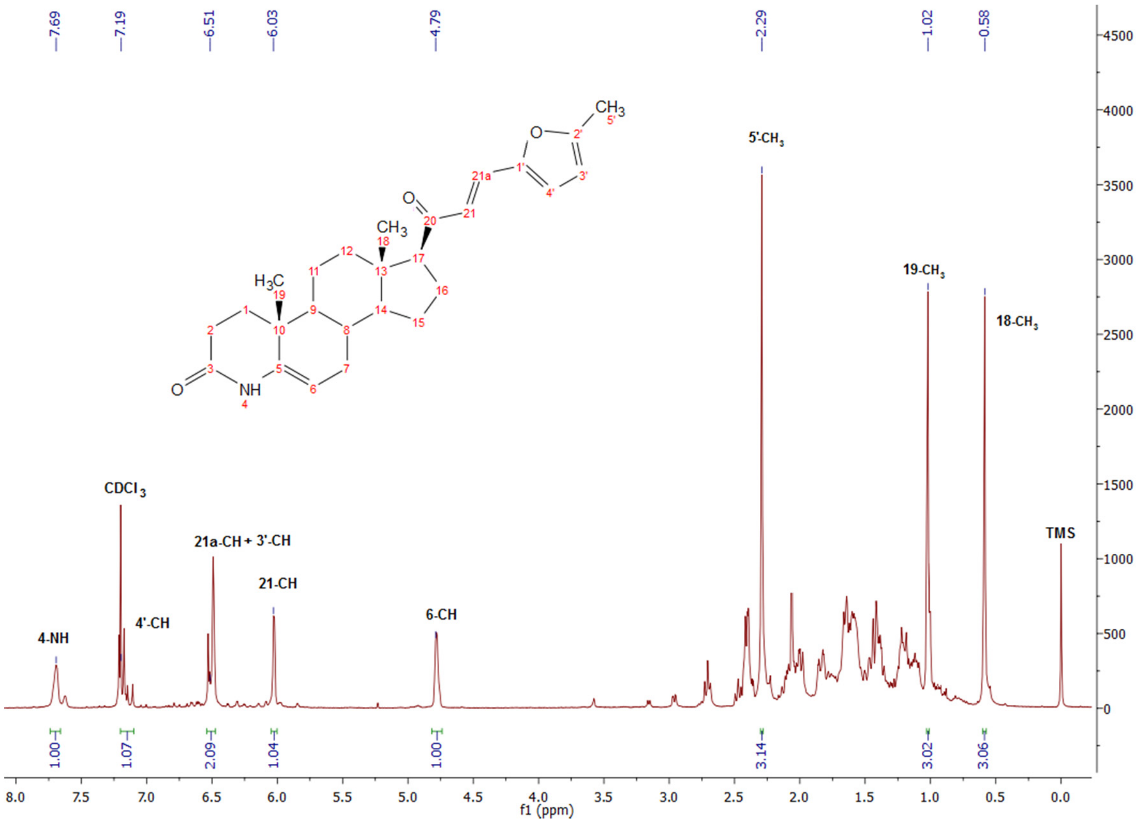

^{13}C NMR spectrum of compound 3f

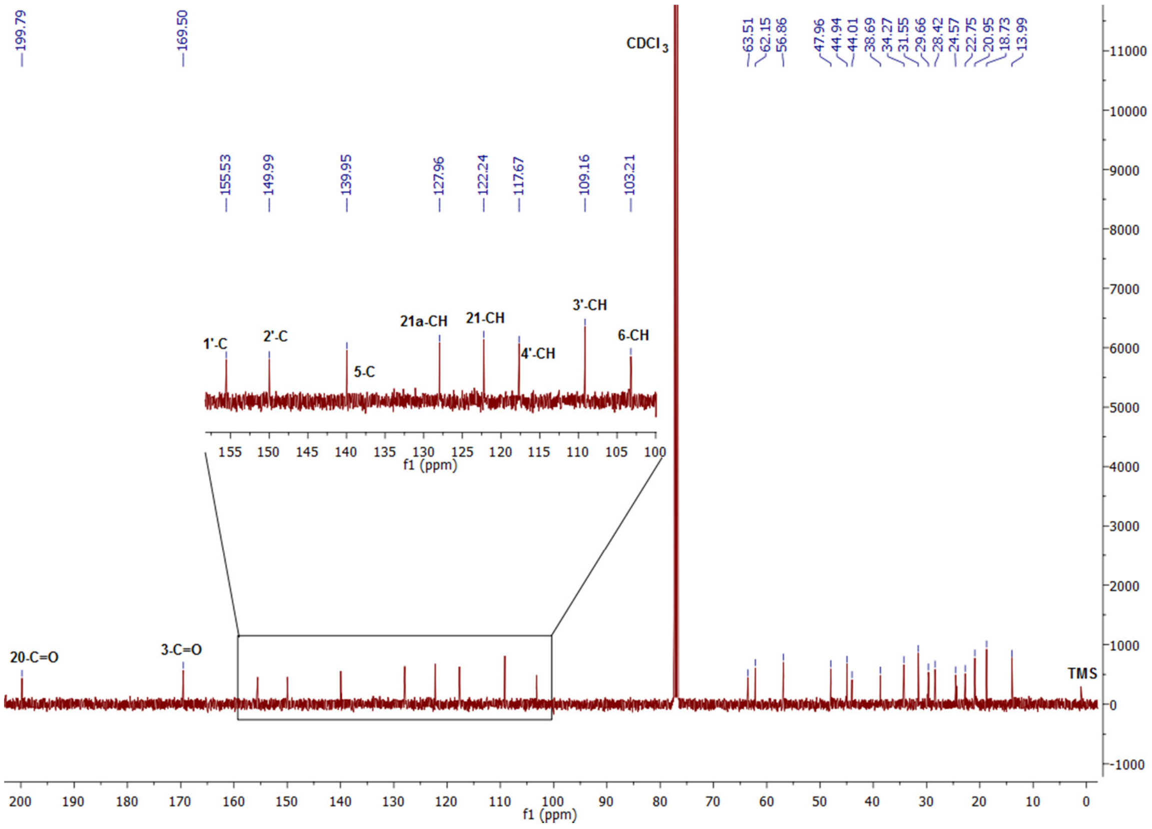

IR spectrum of compound 3f

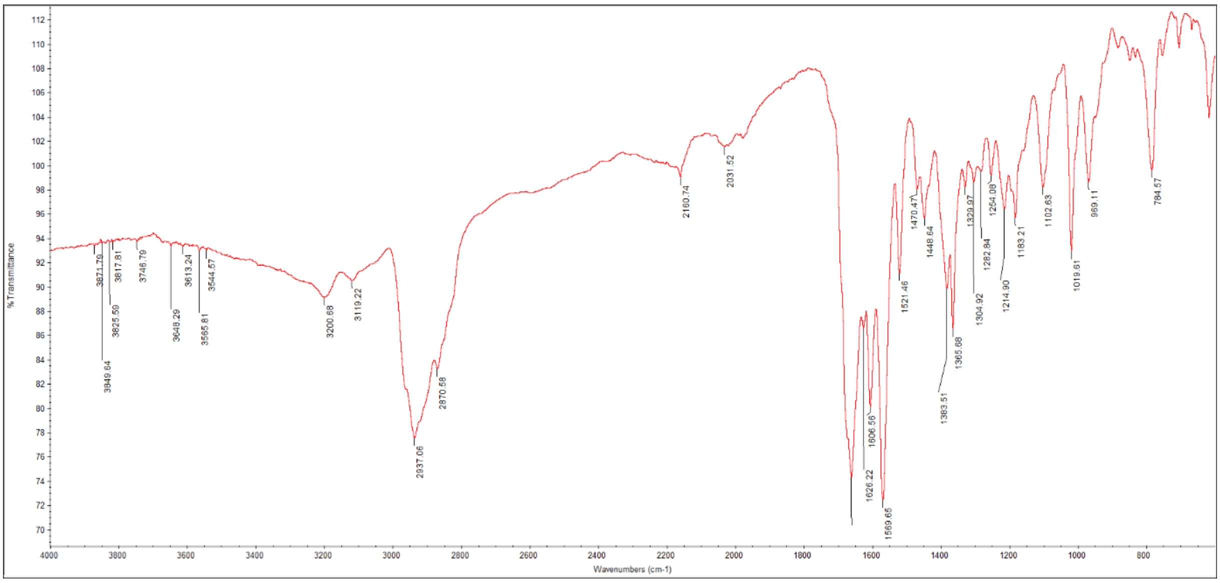

^{1}H NMR spectrum of compound 3g

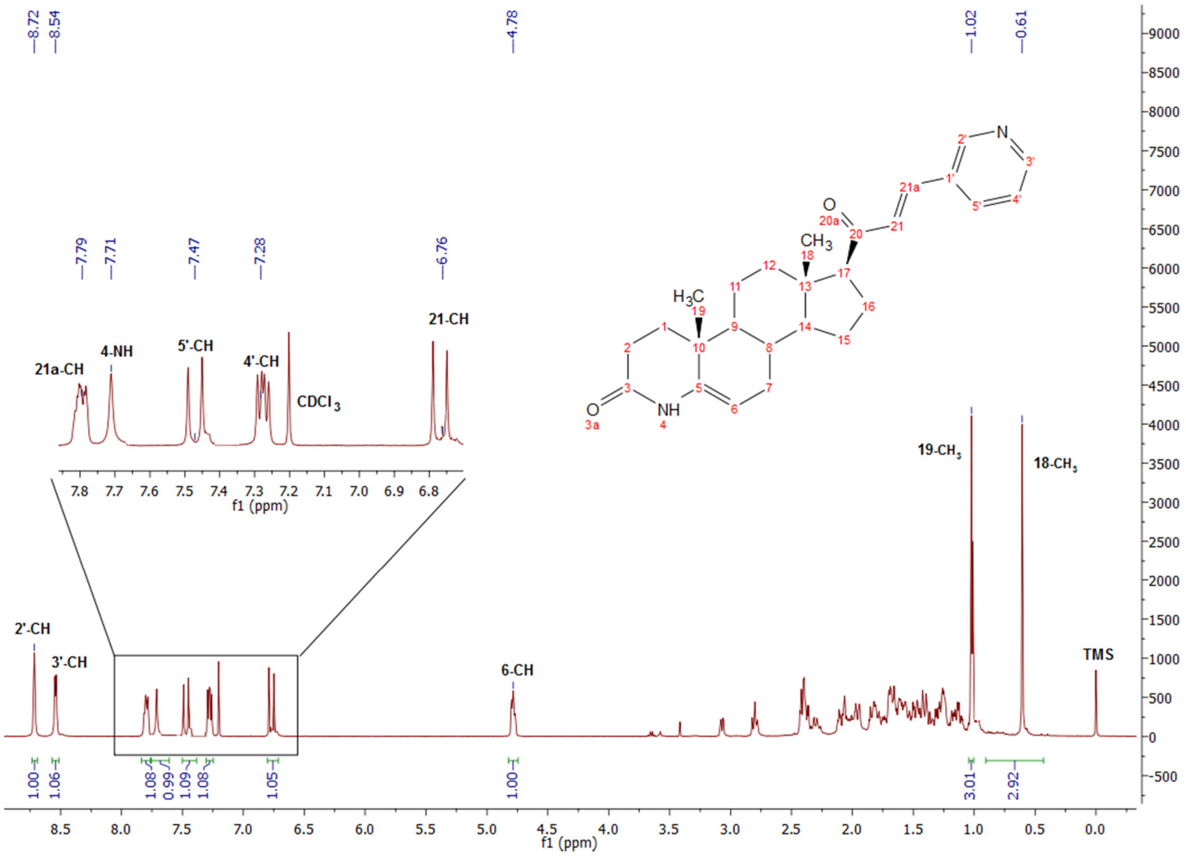

^{13}C NMR spectrum of compound 3g

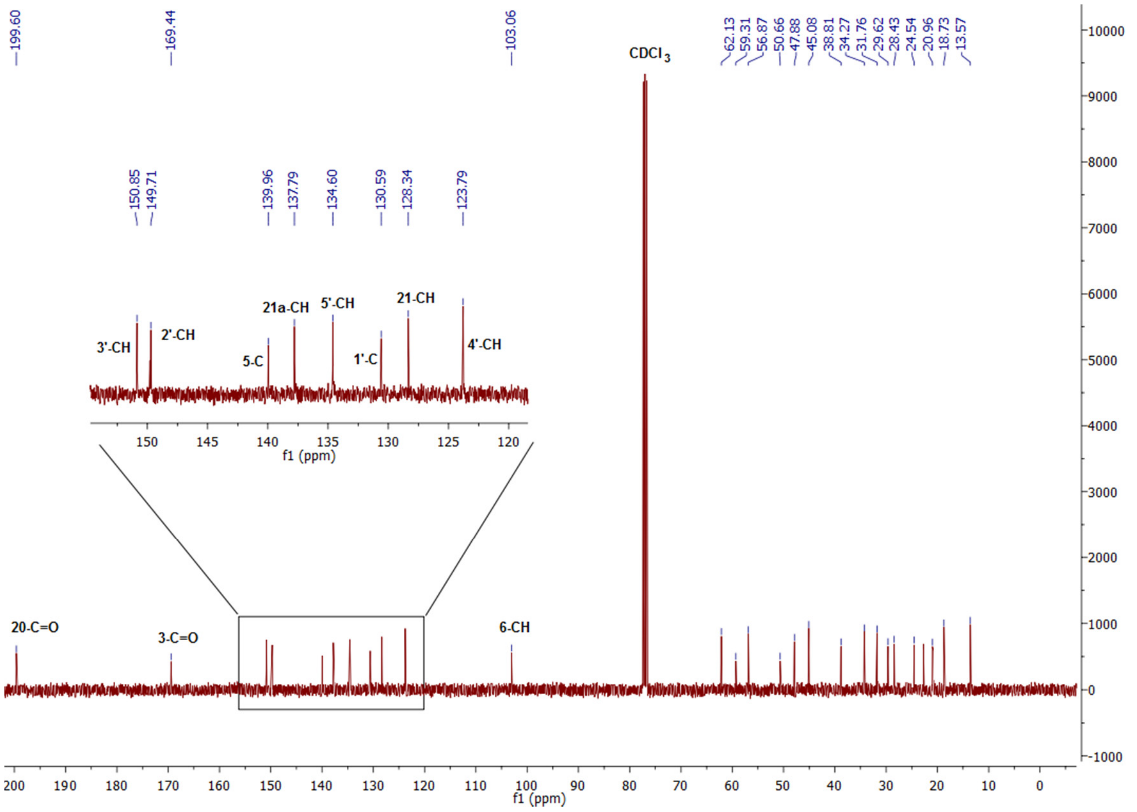

IR spectrum of compound 3g

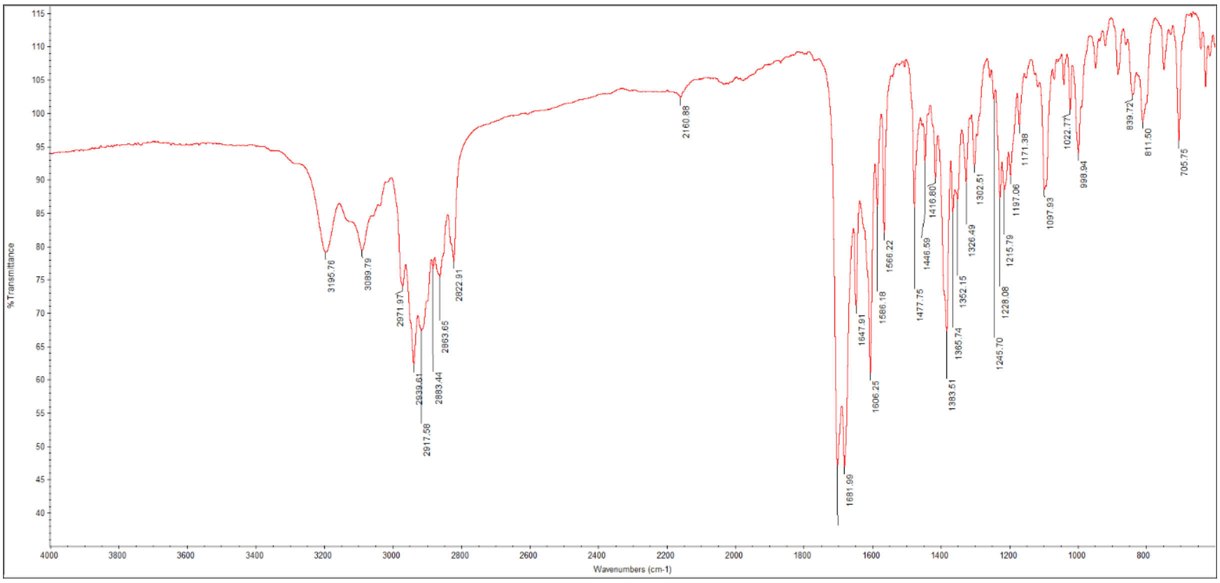

S2. Flow cytometry additional data

Contour plots of flow cytometry assay with propidium iodide staining (for each FACS tube), in LNCaP cells after 24 and 72h of exposition to 50 µM of steroid **3g** and finasteride (and respective controls).

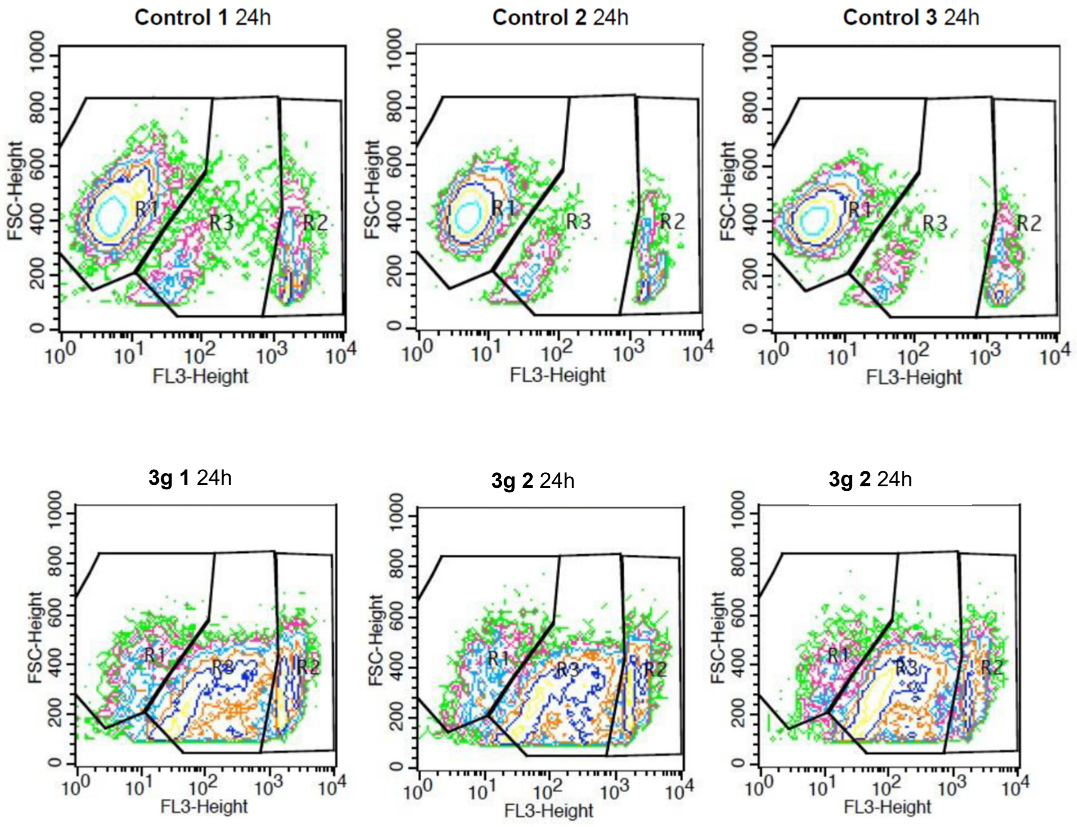

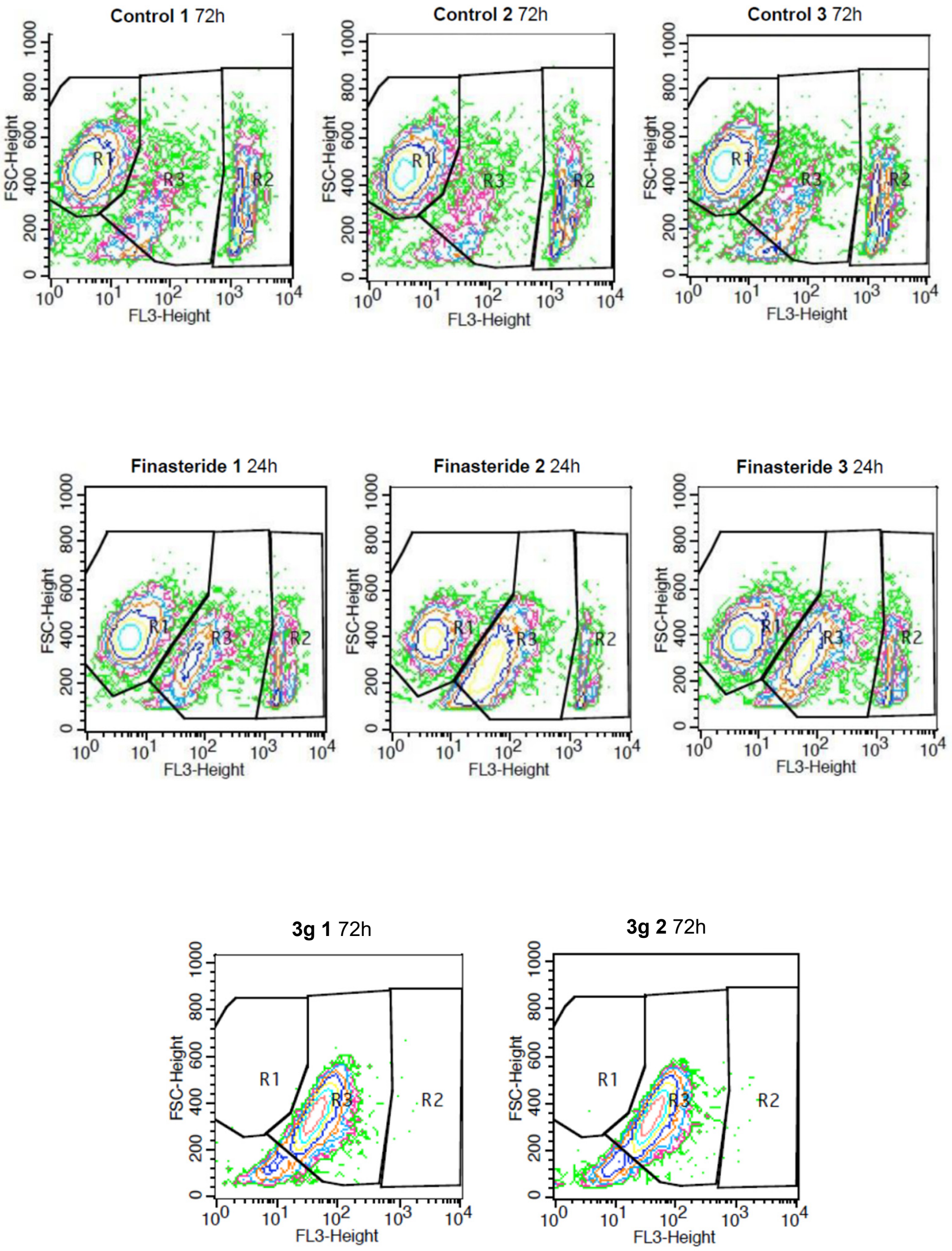
Control 1 72h
Control 2 72h
Control 3 72h
Finasteride 1 24h
Finasteride 2 24h
Finasteride 3 24h
3g 1 72h
3g 2 72h
FSC-Height
FL3-Height
R1
R2
R3
